# The Forgotten Student—Generative AI Use in Medical Education and the Technology-Integrated Educational Design (TIED) Road Map: Scoping Review

**DOI:** 10.2196/96099

**Published:** 2026-10-15

**Authors:** Anita Pienkowska, Olivia Ng, Jianlin Hou, Siew Ping Han

**Affiliations:** 1 Lee Kong Chian School of Medicine, Nanyang Technological University, 11 Mandalay Rd, Singapore, 308232, Singapore, 65 69041267; 2 National Healthcare Group, Singapore, Singapore; 3 Peking University, Beijing, China

**Keywords:** GenAI, generative AI, technology-enabled learning, medical student, Kirkpatrick model, digital educational tools

## Abstract

**Background:**

The growing impact of generative AI (GenAI) on medical education has given rise to many reviews discussing AI literacy, trustworthiness, potential uses, and institutional or faculty benefits. However, there has been surprisingly little review-level attention to its impact on students and learning.

**Objective:**

This scoping review examines the impact of GenAI-enabled educational interventions on undergraduate medical students.

**Methods:**

We followed the PRISMA-ScR (Preferred Reporting Items for Systematic Reviews and Meta-Analyses extension for Scoping Reviews) guidelines. An electronic search was conducted between September 22 and September 24, 2025, across Ovid (MEDLINE and Embase), PubMed, Web of Science, and Scopus, with no language or date restrictions, and excluding reviews, commentaries, letters, editorials, preprints, conference papers, and study protocols. Each record was independently screened by two reviewers, who thoroughly discussed and agreed upon its final inclusion in the analysis. We used a modified version of the Kirkpatrick model as an analytical framework for the final dataset.

**Results:**

Twenty-seven studies were included. Most were published in 2025 (n=21) and in the *Journal of Medical Internet Research* or its sister journals (n=9). The educational interventions described included GenAI used for simulated patient experiences, knowledge or decision support, and content generation. The majority of studies fell under Kirkpatrick level 1 (n=23), focusing on initial user reactions. The studies that fell under Kirkpatrick level 2 (n=16) examined learning outcomes in terms of knowledge gain, skill acquisition, and learning attitudes. No studies were classified as Kirkpatrick level 3. Three studies demonstrated explicit theoretical grounding and therefore were classified as Kirkpatrick level 4 in our modified model. Only 13 studies spanned across levels. Numerous studies fell outside the model, including studies without student-facing interventions (119 studies) and studies focused on GenAI performance (110 studies). Although these studies were not included in the final analysis, they revealed the extent to which other topics dominate the academic conversation on GenAI in medical education.

**Conclusions:**

Our review highlights the volume of studies that prioritize tool performance over learning processes, as well as a trend toward the fragmentation of educational design elements in research evaluating GenAI-enabled educational interventions. Therefore, based on our findings, we developed the Technology-Integrated Educational Design (TIED) road map that reimagines the Kirkpatrick model as a tool to guide student-centered, theory-driven, and iterative design of technology-enabled educational interventions. By integrating preinstructional exploration, mechanism-oriented thinking, educational design, implementation, and evaluation within a single student-centered framework, the road map encourages educators and researchers to consider not only whether technology-enabled interventions work and are acceptable, but also how, for whom, and under what conditions they support meaningful learning.

## Introduction

Amid the rapidly expanding access to AI and generative AI (GenAI)—large language models that can produce text, images, and video—the integration of these technologies into medical education appears inevitable [1,2]. Beyond early reflections on AI literacy needs [3], many reviews focus either on trustworthiness (such as reporting AI performance in medical examinations [4]) and broadly defined effectiveness [5], or on opportunities, challenges, and future directions [6-8]. These accounts primarily propose broad implementation directions and map potential uses, including personalized learning, workload reduction, and learner engagement optimization [6], as well as curriculum development, educational resource optimization, and institutional efficiency gains [9-11]. As such, the literature tends to emphasize institutional or faculty benefits and positions students mainly as users of technology rather than as subjects of learning change.

Surprisingly few reviews explicitly foreground students in discussions, despite students being the primary recipients of educational interventions. One example was a review by Izquierdo-Condoy et al [12] examining the impact of GenAI on cognitive engagement in medical education, finding evidence both of its potential benefits (improved scores on critical thinking, critical awareness, and self-directed learning) and risks (increased cognitive offloading and reduced evaluative judgment). However, only 5 empirical studies were included in their analysis. Similarly, Pham et al [13] analyzed empirical studies to assess the alignment between GenAI use and learning types using Laurillard’s Conversational Framework, finding that GenAI most frequently supports learning through practice (73%), inquiry (70%), production (67%), and acquisition (55%), but far less often through discussion and collaboration (12% each). Their approach focused on how students used GenAI rather than its impact on their learning.

Positioning students at the center of intervention design and evaluation, this review examines what changes occur for learners following GenAI-enabled educational interventions. To address this, we draw on the widely used Kirkpatrick model. First introduced in the 1950s as one of the earliest systematic approaches to evaluating training [14], the model comprises 4 outcome-focused levels: reaction (learners’ responses), learning (acquisition of knowledge, skills, or attitudes), behavior (transfer of learning into practice), and results (broader organizational outcomes, such as improvements in quality, productivity, or efficiency).

Despite critiques—outlined in the Methods section—the Kirkpatrick model remains widely implemented in medical education research [15] because it offers a structured lens for examining change resulting from educational interventions and a clear typology for situating outcomes. Recently, the model has been applied to analyze GenAI-related impacts on learning in health professions education (HPE), including studies examining feedback effects [16], national implementation contexts in Indonesia and China [17], and applications within resident and nursing populations [18]. These studies generally categorized outcomes into Kirkpatrick levels 1 to 4 (corresponding to student reactions, learning outcomes, behavioral change, and broader impacts) without detailed interpretation of what these categories mean and how they apply to HPE contexts. Building on this work, our review focuses specifically on undergraduate (preresidency) medical education and asks what changes occur for students following GenAI-enabled interventions.

## Methods

### Study Design

This study follows scoping review logic and procedures [19] and reports according to the PRISMA-ScR (Preferred Reporting Items for Systematic Reviews and Meta-Analyses extension for Scoping Reviews) and PRISMA-S (Preferred Reporting Items for Systematic Reviews and Meta-Analyses Literature Search Extension) guidelines [20,21] (Checklist 1). This approach is suitable for analyzing a range of studies to describe and map the overall picture of the current scope of the evidence in the field [22]. Given the nature of the study design, ethical approval was not required. Similarly, as per scoping review methodology, we did not formally assess the quality of the included studies [23]. The protocol for the study has not been published or formally registered. An operational workflow, discussed as a team, guided the conduct of the review; consistent with the exploratory and mapping-oriented nature of scoping reviews, aspects of the approach were refined as our understanding of the literature developed. These adaptations, described in the following section, relate primarily to the analytical framework.

### Analytical Framework: Adaptations of the Kirkpatrick Model

#### Rationale for Adapting the Kirkpatrick Model

We selected the Kirkpatrick model as the analytical framework due to its widespread use in medical education and its use in organizing educational outcomes in a straightforward and systematic manner. At the same time, we recognize that the model has been subject to critique; we therefore outline key points, not to dismiss the framework, but to demonstrate awareness of its limitations and to justify our adaptations and analytical decisions. Articulating this rationale is both a matter of conceptual rigor and methodological transparency.

In assessing the use of the Kirkpatrick model in medical education, scholars have noted a few critical issues. The main line of critique concerns the model’s simplicity—also its strength—which may result in an oversimplified representation of complex educational dynamics [24], analyses lacking nuance [25], and assumptions of causal linkages between levels that implicitly privilege higher-level outcomes [26]. In line with these critiques, Allen et al [26] propose alternative program evaluation frameworks that, in their view, better capture the complexity of HPE interventions, such as realist review or the context-input-process-product (CIPP) evaluation.

The decision not to substitute the Kirkpatrick model with an alternative framework reflects our view that evaluation frameworks must be commensurate with the nature of the available evidence, and that many included studies lacked the detailed reporting required by proposed frameworks (nuanced context-mechanism-outcome description for realist evaluations; data on needs assessment prior to the intervention, process, implementation fidelity, cost-effectiveness, or unintended consequences for CIPP-informed analyses). Applying these frameworks would refocus the review and tilt it toward a reflection on the extent of underreporting. Our decision does not imply disagreement with the propositions of Allen et al [26] but is rather a consequence of the nature of current reporting approaches. We did, however, introduce a few modifications to the usual Kirkpatrick model.

#### Reinterpretation of Levels 3 and 4

The Kirkpatrick model was originally developed for business and industrial settings in which training outcomes can be directly linked to behavior (level 3) and organizational impacts (level 4) that are measured in readily available real-world settings. In the context of undergraduate medical education, study authors rarely have access to clinical settings or measurable organizational or patient-level outcomes, as the learning is, by design, preclinical. Moreover, academic research papers are produced within a different communicative and epistemic context from institutional evaluation reports, with a primary aim of advancing knowledge rather than documenting operational impact. A strict reading of behavioral outcomes (level 3) as demonstrated exclusively in clinical settings would be overly restrictive in the context of undergraduate studies. We therefore extended the level 3 interpretation to include behavior enacted consistently and continuously, that is, learning translated into sustained forms of behavior demonstrated continuously rather than through one-off performances, such as a single-instance simulation or an objective structured clinical examination (OSCE).

Similarly, we broadened our interpretation of level 4 by expanding it to “clinical/patient outcomes or pedagogical grounding.” This adaptation involved two conceptual steps. First, we reconsidered what constitutes system-level results in medical education. In their critical analysis of the Kirkpatrick model, Yardley and Dornan [27], drawing on the Best Evidence Medical Education (BEME) collaboration, interpret level 4 as comprising two distinct dimensions: delivery of care or patient outcomes (“Level 4a Change in organizational practice: wider changes in the organization or delivery of care, attributable to an educational program; Level 4b Benefits to patient/clients: any improvement in the health and well-being of patients/clients as a direct result of an educational program” [27]). This interpretation provides greater specificity regarding what “results” may represent within medical training, where improving health care delivery and patient outcomes are the ultimate objectives. However, although such outcomes are theoretically possible, they are unlikely to be routinely measurable in undergraduate, particularly preclinical, medical education, where students have limited opportunities to influence—and for researchers to test—clinical practice. Applying such an interpretation of level 4 would therefore render the category largely unattainable.

As a consequence, we returned to the Kirkpatrick literature itself to examine how “results” are conceptualized. Kirkpatrick and Kirkpatrick [14] argue that one of the most common misconceptions surrounding level 4 arises because “professionals...define results in terms of their small, individual area of the organization instead of globally for the entire company.” They further explain that, particularly in not-for-profit organizations, this means “accomplishing the mission while responsibly using the resources available” [14]. We interpret this as suggesting that level 4 should be understood relative to the mission of the organization rather than through a fixed set of outcomes. Given that undergraduate medical education is delivered within universities, the university’s mission provides the appropriate frame of reference; that is, following John Henry Newman’s description, a university “educates the intellect to reason well in all matters, to reach out towards truth, and to grasp it” [28]. Consequently, initiatives that are explicitly grounded in educational theory and designed to test, refine, or extend pedagogical knowledge contribute to this institutional mission. This interpretation is consistent with arguments that educational theory should function as a mechanism for cumulative knowledge development rather than as a decorative addition to educational research [29]. Furthermore, Kirkpatrick links level 4 evaluation to needs assessment and problem definition, emphasizing alignment between design and outcomes [14], hence suggesting that reflection on level 4 needs to start early in the process.

Accordingly, we reconceptualized level 4 as representing evidence of system-level impact, operationalized either through demonstrable clinical or patient outcomes, or through theoretically grounded educational research that advances pedagogical knowledge. For example, Shalong et al [30] grounded their intervention in self-directed learning theory and evaluated this pedagogical construct (level 4), assessed reactions (level 1), as well as learning outcomes through validated pre-post questionnaires, demonstrating changes in learning (level 2)—no observable behavioral change was evaluated over time (level 3).

#### Beyond the Model

Following another prominent critique of the model—discouraging nuanced analysis of the processes—we decided to add one more level to examine reporting on the mechanisms underpinning the interventions. Its focal question was “What worked, how, and why?” Lastly, we remained attentive to studies excluded from full-text analysis, using them to inform the broader contextual understanding of the field, even when this extended beyond the traditional scope of the Kirkpatrick model.

### Search Strategy, Study Eligibility, and Selection

The population-concept-context framework was defined as follows: the population comprised undergraduate medical students, the concept was the use of GenAI in medical education, and the context was education-related empirical research (Multimedia Appendix 1). We developed and refined the search strategy through iterative testing. An electronic search was conducted between September 22 and September 24, 2025, across Ovid (MEDLINE and Embase), PubMed, Web of Science, and Scopus to maximize coverage. Free-text terms ([“GenAI” OR “generative AI” OR “large language model” OR “gpt” OR “deepseek” OR “LLM”] AND [“medical student” OR “medical education”]) aimed at maximizing coverage were applied to titles and abstracts, with no language or date restrictions.

The search yielded 2316 records. After deduplication, we used TERA (The Evidence Research Accelerator) tools [31] to screen 923 records according to inclusion and exclusion criteria that were thoroughly discussed and agreed upon as a team. Our a priori exclusion criteria comprised (1) reviews, commentaries, letters, and editorials; (2) preprints, conference papers, and study protocols; and (3) studies that did not pertain to undergraduate medical education (ie, postgraduate medical education—residency or continuing professional development; we allowed studies with mixed populations, provided that the population of interest was included). During title and abstract screening, it became apparent that many records concerned the technical performance of GenAI or GenAI use without student-facing interventions. Because the Kirkpatrick model is designed to evaluate educational interventions, it would not be possible to extract relevant information and evaluate these studies within the chosen model. Hence, we refined and tightened the eligibility criteria to include only student-facing intervention studies. As a result, 2 categories of studies were excluded from the final analysis. First, studies evaluating GenAI performance in medical examinations without student participants were excluded, as they focused solely on whether GenAI could simulate student performance. Second, we excluded studies on GenAI in medical education that did not include a student-facing intervention-based study focused on educational student outcomes, for example, studies where content generation was evaluated, or students’ awareness of GenAI tools was studied.

During the title and abstract screening, each researcher independently screened their assigned set of records; we then reached an agreement in pairs (records numbered 1‐462: AP and ON; 463‐923: SPH and JH). To ensure consistent application of the eligibility criteria, the authors (AP, ON, SPH, and JH) met on multiple occasions to discuss the screening approach. We also conducted a dedicated in-person meeting to jointly review a sample of 40 records to test the consistency of the shared interpretation of the eligibility criteria. The team determined that the level of agreement was sufficient to proceed; however, agreement statistics were not formally recorded. This process resulted in 269 eligible records. The tightened inclusion criteria yielded 45 studies to be sought for retrieval. After full-text screening, the final dataset comprised 27 records.

### Data Extraction, Analysis, and Synthesis

Data were extracted in pairs using a collaboratively developed and piloted template and recorded in Microsoft Excel. Following full-text analysis, extracted data were reviewed and finalized through team consensus. Unstructured data were standardized to enable quantitative synthesis, and descriptive statistics were used to summarize key characteristics as well as the Kirkpatrick classification. Data extraction included country, aim, study design, use (purpose) of the GenAI tool, data collection, results and outcomes, reference to pedagogical theory, the Kirkpatrick level, and notes. We allowed for nonexclusive categorization across Kirkpatrick levels; that is, individual studies could be classified under more than one level.

In light of the use of AI for textual data coding observed in our dataset, we needed to take a clear position on how such studies should be classified and what constitutes qualitative analysis. Our classification reflects a stance rooted in qualitative research traditions, in which coding is understood as the beginning rather than the endpoint of qualitative analysis [32,33]. We considered studies to be qualitative or mixed methods only if they demonstrated core qualitative commitments such as interpretive labor, reflexive engagement, contextual meaning-making, and an accountable analytic narrative. In our view, combining descriptive qualitative analysis focused primarily on the summarization and quantification—which already entails limited interpretive accountability—with automated, nonhuman analytic judgment does not demonstrate the interpretive and accountable meaning-making that characterizes qualitative inquiry. We do not dismiss the value of automated topic classification methods; however, none of the studies that relied on semiautomated AI coding in our dataset provided evidence that such interpretive effort had been undertaken; hence, we classified them as quantitative, even if they self-identified as mixed methods, which may be recognized as representing a rather conservative methodological stance [34] within contemporary discussions on automating qualitative research [35,36].

### Ethical Considerations

As a review of previously published literature, this study did not require ethical approval.

## Results

### Study Characteristics

Twenty-seven studies were included in this review (Figure 1), the majority of which (n=21) were published in 2025, with a substantial proportion appearing in the *Journal of Medical Internet Research* and its sister journals (n=9) or in *BMC Medical Education* (n=4). Most studies were conducted in the United States (n=6) and China (n=5), with the remainder from Germany, Japan, Mexico, Sweden, Colombia, France, India, Italy, Turkey, and the United Kingdom. Most studies used quantitative designs (n=23), with 4 studies (2 mixed methods and 2 qualitative studies) [37-40] that used AI to automate qualitative analysis and were therefore not classified as mixed methods.

**Figure 1.**
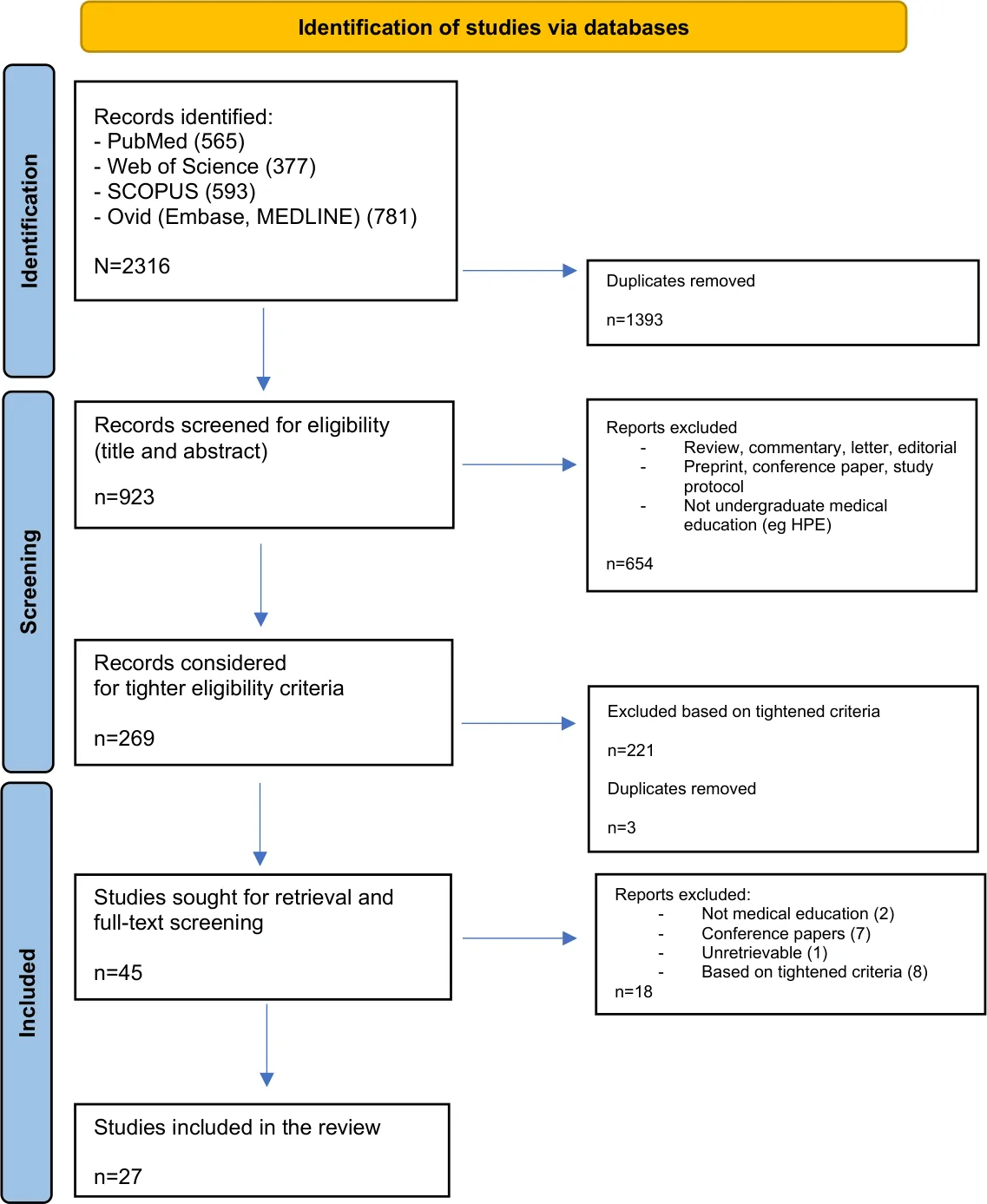
PRISMA-ScR (Preferred Reporting Items for Systematic Reviews and Meta-Analyses extension for Scoping Reviews) flow diagram for the scoping review of the Kirkpatrick classification of generative AI use in medical education. HPE: health professions education.

### GenAI Use

GenAI was used for 3 purposes: simulated patient experience (n=13), knowledge or decision support (n=10), or educational content generation (n=4). Most studies (n=25) used OpenAI’s ChatGPT either as a standalone tool (n=17) or integrated within other platforms or multitool workflows (n=8). Other standalone tools included DeepSeek and Kimi Chat. In a few cases, GenAI tools were used in conjunction with other digital tools, including Stable Diffusion [37,38], DALL·E 3 [41], Leonardo.ai [37], and Whisper-3 [39]. Two studies [37,38] presented particularly sophisticated workflows incorporating tools for plot, image, narration, and audio generation to create videos.

### Kirkpatrick Levels

#### Classification of Included Studies

The Kirkpatrick-focused analysis yielded 23 studies falling under level 1, 16 under level 2, 3 under level 4, and none under level 3 (see Figure 2, illustrating nonexclusive categorization). The studies falling outside the usual Kirkpatrick model—“how,” preinstructional preparatory, and exploratory levels—are described separately. The overview of included studies is presented in Table 1 (an expanded version, with the rationale for level classification, is available in Multimedia Appendix 2).

**Figure 2.**
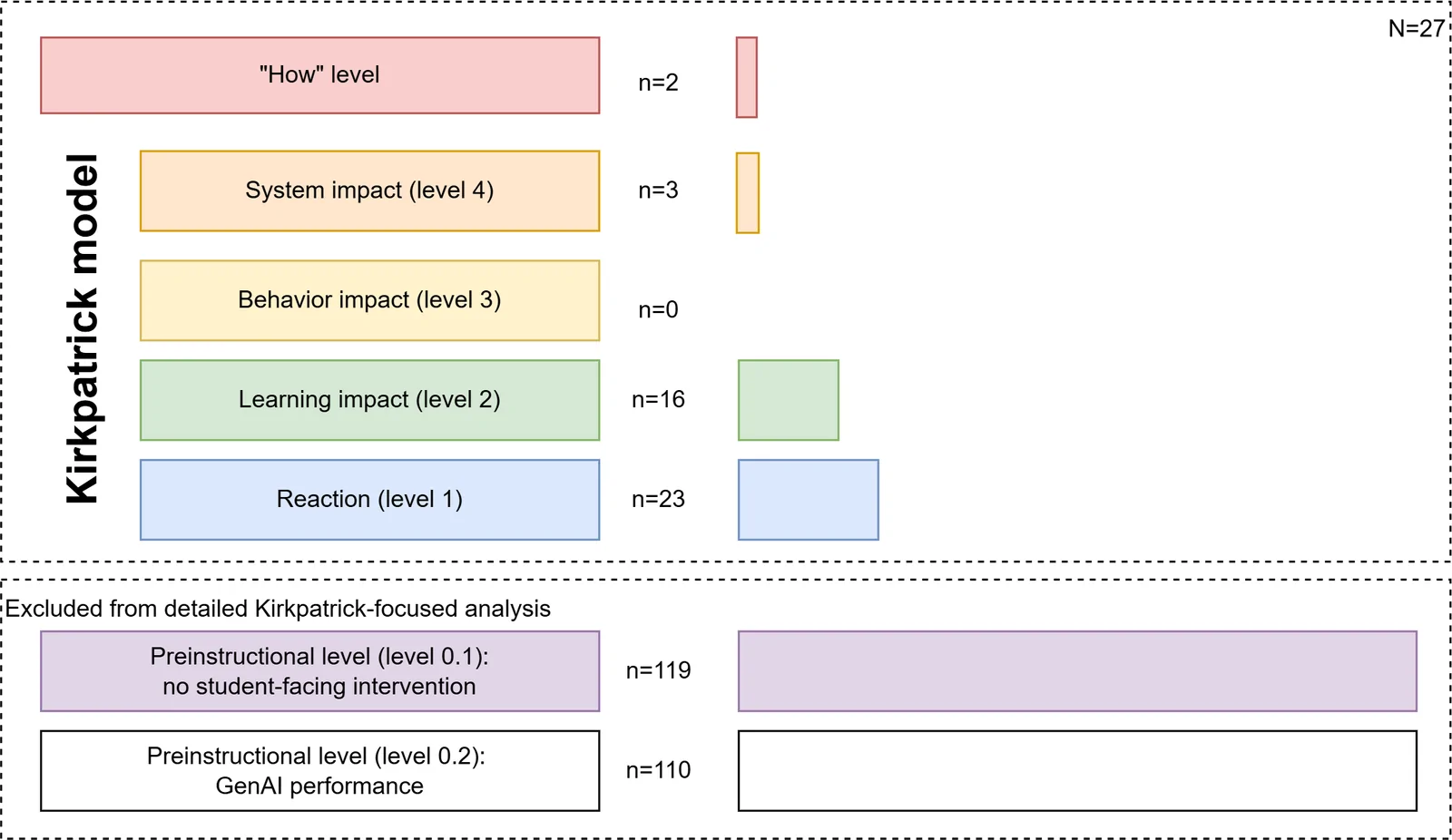
Distribution of studies on generative AI (GenAI) based on the expanded Kirkpatrick levels.

**Table 1.** Kirkpatrick classification of generative AI (GenAI) in medical education: overview of studies.

Included studies			Kirkpatrick levels^a^				
Reference	Use of GenAI	Concise results	Level 1^b^	Level 2^c^	Level 3^d^	Level 4^e^^,^^f^	Level “how”^g^
Amano et al [42]	Knowledge/decision support	Low prior GenAI use; skeptical views toward medical applications but openness to future nonmedical use	✓	✕	✕	✕	✕
Bland 2025 [37]	Educational content generation	Preference for GenAI-enhanced content over traditional teaching, with high scores across situational interest dimensions	✓	✕	✕	✕	✕
Borg et al 2024 [43]	Simulated patient	More authentic and interactive learning experience compared to the conventional computer-based platforms. Three main themes were identified: authenticity, virtual patient application, and strengths and limitations	✓	✕	✕	✕	✕
Ebihara et al [44]	Knowledge/decision support	Positive perceptions; valued for clarity and support, and used mainly for workload and communication rather than emotional stress	✓	✕	✕	✕	✕
Holderried et al [40]	Simulated patient	Overall positive user experience	✓	✕	✕	✕	✕
Laverde et al [45]	Simulated patient	Overall positive user experience	✓	✕	✕	✕	✕
Liu et al [46]	Simulated patient	Helpful; expression of a desire to use it again in the future	✓	✕	✕	✕	✕
Peralta Ramirez et al [47]	Simulated patient	High satisfaction (clarity, appropriateness, and usefulness)	✓	✕	✕	✕	✕
Tekin et al [48]	Educational content generation	Good test performance; clear GenAI outputs and high interest in retaking the examination	✓	✕	✕	✕	✕
Thesen et al [39]	Simulated patient	Positive experiences with flexible, low-pressure learning and real-time feedback; authenticity limitations noted	✓	✕	✕	✕	✕
Borg et al 2025 [49]	Simulated patient	Positive perceptions; themes included realism, immersion, skill development, and procedural limitations	✓	✓	✕	✕	✕
Dupont et al [50]	Simulated patient	Significantly higher OSCE^h^ scores in the intervention group	✓	✓	✕	✕	✕
Hassoulas et al [51]	Simulated patient	Better performance in formative assessments	✓	✓	✕	✕	✕
Li et al [52]	Knowledge/decision support	Higher scores and faster completion; effective for basic knowledge but limited for complex reasoning, with concerns about reduced interaction and homogenized learning	✓	✓	✕	✕	✕
Mashburn et al [53]	Knowledge/decision support	Greater knowledge gains among women; no gender differences in accuracy or satisfaction	✓	✓	✕	✕	✕
Suárez-García et al [54]	Simulated patient	Improved performance; lower baseline empathy and higher digital self-efficacy predicted higher improvement	✓	✓	✕	✕	✕
Wang et al [55]	Simulated patient	Significantly higher posttraining scores with improved motivation, communication skills, and satisfaction	✓	✓	✕	✕	✕
Weuthen et al [56]	Knowledge/decision support	Better performance; increase in self-assessment with regard to specialist knowledge	✓	✓	✕	✕	✕
Worthley et al [38]	Educational content generation	Better performance; improved engagement and interest; preference over other formats	✓	✓	✕	✕	✕
Yamamoto et al [57]	Simulated patient	Significantly higher scores in medical interviews	✓	✓	✕	✕	✕
Brügge et al [58]	Simulated patient	Better performance based on clinical reasoning indicator	✕	✓	✕	✕	✕
Gan et al [59]	Knowledge/decision support	Better performance	✕	✓	✕	✕	✕
Montagna et al [60]	Knowledge/decision support	Faster answering time, but the clinical practice guidance group performed best in nearly all domains	✕	✓	✕	✕	✕
Alomar et al [41]	Educational content generation	Stable performance over time; higher situational interest	✓	✓	✕	✓	✕
Shalong et al [30]	Knowledge/decision support	Sustained improvements in self-directed learning and critical thinking over time	✓	✓	✕	✓	✕
Kalam et al [61]	Knowledge/decision support	Initial performance gains were not maintained, with no differences in retention at 1-week follow-up	✓	✓	✕	✓	✓
Skryd and Lawrence [62]	Knowledge/decision support	Themes: discrete medical knowledge inquiries, building differential diagnoses and engaging dual process thinking, challenging medical axioms, using cognitive aids to support acute care decision-making, and facilitating conversations with subspecialties and others	✕	✕	✕	✕	✓

a ✓ indicates the characteristic was present; ✕ indicates it was absent.

b Level 1: reaction.

c Level 2: learning impact.

d Level 3: behavior impact (behavior enacted consistently and continuously).

e Level 4: system impact (clinical/patient outcomes or pedagogical grounding).

f Level 4 was adapted as described in the Methods section and is therefore not directly comparable with level 4 in conventional applications of the Kirkpatrick framework.

g Level “how”: proposed additional level for conceptually comprehensive examinations of educational impact.

h OSCE: objective structured clinical examination.

#### Level 1: Reaction

A total of 23 studies addressed Kirkpatrick level 1, which focuses on learners’ reactions to an educational experience, typically measured through satisfaction [47], perception [50,61], or engagement [41]. The majority of these studies explored the perceived utility of GenAI tools in supporting clinical reasoning. Interventions included virtual patient platforms [39,40,43,45,47,49,51], AI-supported mentoring [44], history-taking exercises [46,55], and OSCE preparation [50,57]. Several of these studies also leveraged GenAI’s capability for automated feedback generation platforms [39,46,47]. Additional applications included the use of GenAI as a study resource or teaching assistant [30,42,52,56,61], question-generation tools [48], narrative-based short case videos [37,38], as well as other functions, such as answering clinical queries [53] and generating diagnostic images [41].

Students reported that GenAI-generated questions were clear [48], and satisfaction surveys consistently indicated high levels of satisfaction and favorable impressions of GenAI-generated outputs [40,46,55,56]. Several studies also noted that learners perceived a greater sense of authenticity when engaging with GenAI tools [41,43]. Additionally, some studies reported associations with self-reported improvements in self-directed learning [30], as well as with the integration of GenAI with other digital technologies to support simulation-based education [45,49]. These positive self-reported reactions largely reflect an initial response to the tool. However, it is equally important to examine whether meaningful learning occurred and which specific educational interventions supported it.

#### Level 2: Learning

Sixteen studies examined learning outcomes, including knowledge recall or application, skill acquisition, learning attitudes, and combinations of these. Of the 7 studies focusing on knowledge gain, 4 assessed this based on multiple-choice questions (MCQs) testing recall of content in examinations or quizzes [38,41,59,61]. The other 3 studies tested knowledge application through case studies with open-ended questions [60] or a mixture of open-ended and MCQs [53,56]. Of the 6 studies focusing on skill acquisition (eg, clinical reasoning and communication skills), 3 assessed this through OSCEs or their equivalent [50,55,57], 2 assessed this through interactions with human-simulated patients [54] or ChatGPT-simulated patients [58], while the remaining study surveyed participants on their self-perceived improvements in skill levels [49]. Three studies explored a mix of learning outcomes. Shalong et al [30] examined both changes in learning attitudes, in terms of self-directed learning, and improvements in critical thinking skills. Hassoulas et al [51] assessed both knowledge recall through MCQ quizzes and learning attitudes in terms of self-reported confidence in scientific knowledge, clinical competence, and opinions of tech-enabled learning. Finally, Li et al [52] looked at all 3 domains through examination performance (knowledge), speed of case study completion (skill), and perceptions of AI tools in learning (attitude).

All but 3 studies reported “significant” improvements [56,59,60]. Some studies lacked a control group [54,57,61], while other studies examined the effect of GenAI integrated with other educational technologies [38,49,51], so it was not possible to separate the effects of GenAI from other aspects of the intervention. Five studies used a randomized controlled trial study design where the GenAI-based intervention was compared with non–GenAI-based but equivalent educational activities [30,52,55,58,59].

#### Level 3: Behavior

No studies were classified as examining behavioral outcomes in clinical settings or behavior enacted consistently and continuously. Several studies used GenAI tools to support the development or assessment of skills that held the potential for impacting behavior, including history-taking [40,45,46,49,55], communication [50,54], clinical decision-making [51,58], and medical interviewing [57]. However, this potential was not realized analytically, as studies focused on learner reactions [40,45,46] or knowledge gains only [51,58], or assessed isolated or short-term performance—such as through one-off assessments [54,57] or pre-post OSCE evaluations [50,55]—rather than sustained behavioral change, indicating assessment of skill acquisition rather than behavioral outcomes.

#### Level 4: System-Level Outcomes

Our expanded interpretation of Kirkpatrick level 4 allowed us to include 3 studies that demonstrated explicit theoretical grounding, with theory actively used in the discussion and a clear alignment between theoretical assumptions, study design, and interpretation of findings. Shalong et al [30] examined self-directed learning; Kalam et al [61] grounded their GenAI intervention explicitly in cognitive load theory; Alomar et al [41] discussed mnemonic techniques grounded in learning theory even though the integration of GenAI itself was not explicitly theorized. By grounding the intervention, evaluation, and discussion in theory, these studies introduced insights into student learning impacted by GenAI.

Several studies represented missed opportunities for deeper theoretical engagement, particularly those using simulation-based approaches without explicit pedagogical grounding or analysis of learning processes [40,43,45-47,49,54,55,57,58]. Other studies referenced educational concepts only superficially—for example, technology-enhanced learning or the technology acceptance model—thereby using theory to inform tool development but not the interpretation of findings [44,51].

#### Beyond Kirkpatrick (Mechanism Level: “How”)

The most in-depth examination of how GenAI shapes users’ learning was provided by 2 studies: Skryd and Lawrence [62] and Kalam et al [61]. In a retrospective qualitative study of medical students using a GenAI-based decision support system in hospital wards, Skryd and Lawrence [62] demonstrated a wide range of cognitive and practical processes and functions. Kalam et al [61], on the other hand, discussed GenAI integration through the lens of cognitive load theory, highlighting the mechanisms by which GPT-based support reduces extraneous cognitive load and contributes to short-term learning gains.

A few studies partially engaged in describing the mechanisms of impact but did not answer the question of how GenAI specifically contributed to or transformed these pedagogical mechanisms [41,54,58]. Notably, most studies engaged extensively with how the tool was built rather than how learning occurred, providing detailed accounts of system development and technical implementation [37,40,45,48].

#### Preinstructional Levels

##### Level 0.1: No Student-Facing Intervention

A substantial number of studies did not involve students as participants in an educational intervention. Consequently, these studies fell outside the Kirkpatrick model. This group comprised 119 reports (see Multimedia Appendix 3 for a detailed list) which, although situated within medical education, focused primarily on the technical capabilities of GenAI (eg, content generation) or on students’ general awareness, acceptance, or attitudes toward GenAI, rather than on its use within specific educational interventions. As these studies did not examine specific learning processes impacting student learning and educational outcomes, they were excluded from the final dataset. Nevertheless, given their prevalence, we highlight this category to contextualize the broader research landscape.

##### Level 0.2: GenAI Performance

A second, substantial body of literature evaluated the performance of GenAI in tests, examinations, or national medical licensing assessments. Of the 269 papers identified at the preliminary screening phase, 110 studies (see Multimedia Appendix 3 for a detailed list) focused exclusively on GenAI performance without involving students as participants or examining their experiences, preferences, or learning processes. The prominence of this category suggests a particular orientation within the field—potentially reflecting an emphasis on technical benchmarking or precautionary concerns regarding tool safety prior to educational implementation. Regardless of the underlying motivation, the absence of student involvement meant that these studies did not meet the inclusion criteria but concurrently displayed a distinct trend in knowledge production in the field.

### Level Overlap

Of the 27 included studies, 14 were classified within a single Kirkpatrick category, whereas 13 spanned multiple levels. Ten studies were positioned across levels 1 and 2, examining both learner reactions and learning outcomes [38,49-57]. Two studies spanned levels 1, 2, and 4 [30,41], and one extended further to include the additional “how” category [61], representing the most conceptually comprehensive examination of educational impact.

## Discussion

### Primary Findings

Overall, the Kirkpatrick-focused analysis revealed a strong concentration of studies on learner reactions and learning outcomes, with 13 spanning across levels. Beyond the Kirkpatrick framework, a substantial body of literature fell outside educational intervention evaluation altogether: 119 studies did not report on student-facing interventions, and a further 110 focused exclusively on GenAI performance in assessments or examinations. Studies at level 1 consistently reported positive learner perceptions and satisfaction with GenAI-supported tools, while level 2 studies generally suggested improvements in learning outcomes. Notably, very few studies examined how GenAI impacted learners or were theoretically grounded.

### Comparison With Prior Work

The predominance of studies reporting outcomes at the reaction and learning levels, with few addressing behavioral or system-level outcomes (levels 3 and 4), should not be viewed as an anomaly. Similar patterns have been documented not only in GenAI research [16,18], but also across studies of other educational technologies, such as virtual simulation [63], and instructional approaches including team-based learning [64]. Prior reviews suggest that evidence at levels 3 and 4 tends to emerge when technologies are examined over extended periods—for example, in a study of web-based continuing medical education [65], technology-enhanced oncology education over a decade [66], or e-learning in undergraduate clinical education over two decades [67]. These observations indicate that levels 3 and 4 outcomes may depend on the duration and extent of technology integration. Given the cyclical emergence of new educational technologies, with attention frequently shifting to successive innovations [2], the low prominence or absence of levels 3 and 4 outcomes, understood as outcomes in clinical settings, is therefore expected; however, in light of the broadened definitions introduced in this review (sustained behavior for level 3 and theoretical grounding for level 4), their low representation points toward a deeper issue in how the field designs and studies educational interventions.

### The Forgotten Medical Student: Students as Peripheral Analytic Participants

Despite being situated within medical education, a substantial proportion of studies displace students from the center of inquiry in 2 key ways. First, there is limited use of explicit theoretical grounding; second, many studies focus on what GenAI can do for educators or how it performs. The marginalization of medical students is not new. Ludmerer [68] described a similar dynamic resulting from the expansion of biomedical research and clinical service, which produced what he termed “the forgotten medical student.”

In our analysis, we draw attention to the volume of studies that prioritize tool performance over learning processes, thereby directing attention away from how students learn with, through, or alongside GenAI. In doing so, GenAI research risks repeating historical cycles of educational technology enthusiasm without generating durable pedagogical insight. Our findings mirror those of Aster et al [69], who reported that 52% of studies focused primarily on demonstrating GenAI tool performance rather than educational impact. Similarly, the limited presence of explicit theoretical grounding reflects broader trends in medical education research [18,29], despite theory’s importance in explaining why technology-enabled interventions succeed or fail [29]. A related concern is the tendency to treat Kirkpatrick levels as disconnected evaluative categories rather than interconnected elements of educational design. Few reviews report studies spanning multiple levels [70]. These findings reinforce fragmentation across design, theory, and evaluation, as well as insufficient foregrounding of students at the outset of research design. We therefore argue that the key issue is not insufficient outcomes but insufficient integration across design, theory, and evaluation, resulting in fragmentation rather than integration of steps, which results in the systemic marginalization of students as analytic participants.

### From Fragmentation to Integration

We therefore propose the Technology-Integrated Educational Design (TIED) road map, a fresh look at the Kirkpatrick model that encourages the integration of steps across educational design, implementation, and evaluation (rather than treating levels as discrete categories for evaluation purposes only), while also showing the often-overlooked preinstructional stages of educational innovation. The TIED road map is intended as a reminder to reposition students at the center of the intervention lifecycle (Figure 3).

**Figure 3.**
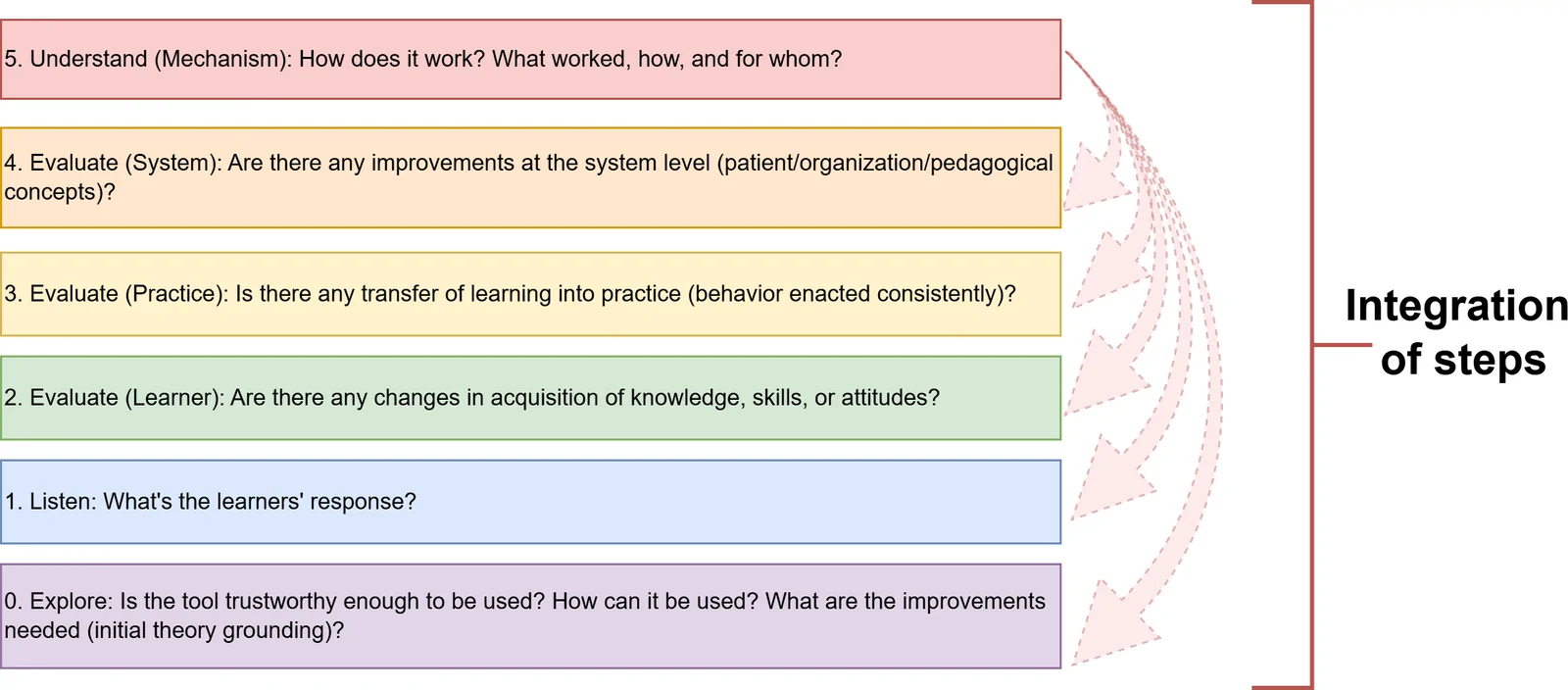
The Kirkpatrick model applied as an educational design road map: Technology-Integrated Educational Design (TIED) road map.

An integrated design begins with preinstructional exploration, addressing questions of trustworthiness, appropriateness, and learner needs: *Is the tool suitable for educational use? How can it meaningfully support learning? What needs will it meet?* This exploratory phase anticipates downstream learning, behavioral, and system-level implications and is grounded in theoretical considerations from the outset. Subsequent stages involve examining learner responses; evaluating changes in knowledge, skills, and attitudes; and assessing transfer into sustained practice. Crucially, the logic also requires mechanism-oriented inquiry that examines how GenAI functions pedagogically—what works, for whom, and under what conditions.

Rather than sequential checkpoints, these steps should inform design, implementation, and evaluation continuously and iteratively. Without such integration, research risks reproducing fragmented, single-level studies that prioritize tool performance or isolated outcomes while marginalizing students as active participants in learning. The TIED road map serves as a reminder to maintain student centrality and support cumulative, theory-informed knowledge development in GenAI-enabled medical education.

### Contributions

The TIED road map has not been conceived in a vacuum but builds on previous work in educational evaluation, instructional design, and user-centered digital innovation. We believe its contributions lie in 4 main areas. First, it extends the New World Kirkpatrick Model [14], which advocates working backwards from desired system-level outcomes. In doing so, it reinforces the original intention of the New World Kirkpatrick Model by encouraging its use not only as an evaluation framework but also as a design framework. Moreover, the TIED road map adopts this principle of backward design while concurrently redefining levels 3 and 4 (Evaluate stages for System and Practice) for undergraduate medical education. In doing so, it encourages educators and researchers to reflect early in the design process on what learner behaviors enacted consistently would look like and how these behaviors might contribute to broader pedagogical impact.

Second, TIED explicitly incorporates mechanism-oriented thinking. The road map responds to previous critiques of the Kirkpatrick model, which have argued that it offers limited guidance on evaluating intervention mechanisms [24,25]. TIED explicitly incorporates a mechanism-oriented step, encouraging early and iterative reflection on *how* interventions produce educational outcomes.

Third, TIED makes the preinstructional phase explicit. The Explore stage aligns closely with human-centered design approaches in [71] by emphasizing the exploration of learner needs alongside experimentation with the capabilities, trustworthiness, and appropriateness of technology. Similarly, the Listen stage reflects formative usability and user experience evaluation and parallels digital innovation frameworks, such as the IDEAS (integrate, design, assess, and share) framework for digital interventions, which emphasize iterative user feedback and refinement throughout the design process [72]. Rather than treating these activities as separate design exercises, TIED incorporates them into the educational intervention lifecycle, explicitly linking educational design, educational evaluation, and human-computer interaction.

Finally, the principal contribution of TIED lies in integrating these perspectives within a single road map. As Susan Gasson argued, user-centered systems development can fail to promote human interests when it becomes dominated by technical goals rather than the people it is intended to serve [73]. Similarly, educational technology research may risk treating needs assessment, intervention design, implementation, mechanisms of learning, and evaluation as isolated activities. By bringing these elements together within a single iterative road map, TIED positions students—not the technology or the evaluation metrics—as the central reference point throughout the educational intervention lifecycle.

### Limitations

This review has several strengths, alongside important limitations. Given the rapid pace of publication in GenAI, additional relevant studies may have emerged after the September 2025 search, limiting the comprehensiveness of the evidence mapped in this review. We also acknowledge that expanding the scope of the review beyond undergraduate medical education to include graduate, in-training, and other professional education contexts could have provided a more robust and comparative analysis.

Anticipating critiques aligned with Yardley and Dornan’s [27] argument that the Kirkpatrick framework rests on assumptions best suited to relatively simple instructional designs and short-term outcomes, we acknowledge that alternative analytical approaches could yield more action-oriented recommendations for educators and researchers. However, given that the field of GenAI in medical education remains in an early, exploratory phase, we deliberately focused on identifying the most salient characteristics and dominant trends within the existing scholarship. Our aim was not to prescribe detailed suggestions for practice but to provide a foundation for broader reflection and to encourage a shift in prevailing research orientations.

### Conclusions

This review demonstrates that research on GenAI in medical education is currently concentrated on learner reactions, learning outcomes, and tool performance, with limited attention to learner-centered processes and mechanisms. We argue that there is a pressing need for integrated educational design that connects preinstructional exploration, learning processes and theory, and evaluative considerations across stages. We propose the TIED road map, which builds on established work in educational evaluation, instructional design, and human-centered digital innovation. By integrating preinstructional exploration, mechanism-oriented thinking, and backward design with redefined levels 3 and 4 within a single student-centered framework, the road map encourages educators and researchers to consider not only whether technology-enabled interventions work but also how, for whom, and under what conditions they support meaningful learning.

## Supplementary material

10.2196/96099 Multimedia Appendix 1 Search strategy.

10.2196/96099 Multimedia Appendix 2 Summary of papers.

10.2196/96099 Multimedia Appendix 3 Level 0 papers.

10.2196/96099 Checklist 1 PRISMA-ScR and PRISMA-S guidelines.

## Abbreviations

BEME: Best Evidence Medical Education
CIPP: context-input-process-product
GenAI: generative AI
HPE: health professions education
IDEAS: integrate, design, assess, and share
MCQ: multiple-choice question
OSCE: objective structured clinical examination
PRISMA-S: Preferred Reporting Items for Systematic Reviews and Meta-Analyses Literature Search Extension
PRISMA-ScR: Preferred Reporting Items for Systematic Reviews and Meta-Analyses extension for Scoping Reviews
TERA: The Evidence Research Accelerator
TIED: Technology-Integrated Educational Design

## References

[1] Zhang Y, Xie X, Xu Q (2025). ChatGPT in medical education: bibliometric and visual analysis. JMIR Med Educ.

[2] Li R, Wu T (2025). Evolution of artificial intelligence in medical education from 2000 to 2024: bibliometric analysis. Interact J Med Res.

[3] Civaner MM, Uncu Y, Bulut F, Chalil EG, Tatli A (2022). Artificial intelligence in medical education: a cross-sectional needs assessment. BMC Med Educ.

[4] Brin D, Sorin V, Konen E, Nadkarni G, Glicksberg BS, Klang E (2024). How GPT models perform on the United States medical licensing examination: a systematic review. Discov Appl Sci.

[5] Li J, Yin K, Wang Y, Jiang X, Chen D (2025). Effectiveness of generative artificial intelligence-based teaching versus traditional teaching methods in medical education: a meta-analysis of randomized controlled trials. BMC Med Educ.

[6] Adarkwah MA, Badu SA, Osei EA, Adu-Gyamfi E, Odame J, Schneider K (2025). ChatGPT in healthcare education: a double-edged sword of trends, challenges, and opportunities. Discov Educ.

[7] Lucas HC, Upperman JS, Robinson JR (2024). A systematic review of large language models and their implications in medical education. Med Educ.

[8] Preiksaitis C, Rose C (2023). Opportunities, challenges, and future directions of generative artificial intelligence in medical education: scoping review. JMIR Med Educ.

[9] Furey P, Town A, Sumera K, Webster CA (2024). Approaches for integrating generative artificial intelligence in emergency healthcare education within higher education: a scoping review. Crit Care Innov.

[10] Astbury H, Fortune E, Ponte CD, Lyons K, Shaw L (2026). Generative artificial intelligence for teaching and assessment in health professions education: a scoping review. Nurse Educ Pract.

[11] Lin Y, Luo Z, Ye Z (2025). Applications, challenges, and prospects of generative artificial intelligence empowering medical education: scoping review. JMIR Med Educ.

[12] Izquierdo-Condoy JS, Arias-Intriago M, Tello-De-la-Torre A, Busch F, Ortiz-Prado E (2025). Generative artificial intelligence in medical education: enhancing critical thinking or undermining cognitive autonomy?. J Med Internet Res.

[13] Pham TD, Karunaratne N, Exintaris B (2025). The impact of generative AI on health professional education: a systematic review in the context of student learning. Med Educ.

[14] Kirkpatrick JD, Kirkpatrick WK (2016). Kirkpatrick’s Four Levels of Training Evaluation.

[15] Alsalamah A, Callinan C (2021). The Kirkpatrick model for training evaluation: bibliometric analysis after 60 years (1959–2020). Ind Commer Train.

[16] Kıyak YS, İş-Kara T, Emekli E (2026). Applications and outcomes of large-language-model-generated feedback in undergraduate medical education: a scoping review. Med Sci Educ.

[17] Utomo PS, Yan J (2025). The use of GenAI in medical education in China and Indonesia: a comparative literature review. Educ Health.

[18] Feigerlova E, Hani H, Hothersall-Davies E (2025). A systematic review of the impact of artificial intelligence on educational outcomes in health professions education. BMC Med Educ.

[19] Peters MDJ, Godfrey CM, Khalil H, McInerney P, Parker D, Soares CB (2015). Guidance for conducting systematic scoping reviews. Int J Evid Based Healthc.

[20] Tricco AC, Lillie E, Zarin W (2018). PRISMA extension for Scoping Reviews (PRISMA-ScR): checklist and explanation. Ann Intern Med.

[21] Rethlefsen ML, Kirtley S, Waffenschmidt S (2021). PRISMA-S: an extension to the PRISMA statement for reporting literature searches in systematic reviews. Syst Rev.

[22] Munn Z, Peters MDJ, Stern C, Tufanaru C, McArthur A, Aromataris E (2018). Systematic review or scoping review? Guidance for authors when choosing between a systematic or scoping review approach. BMC Med Res Methodol.

[23] Arksey H, O’Malley L (2005). Scoping studies: towards a methodological framework. Int J Soc Res Methodol.

[24] Cahapay M (2021). Kirkpatrick model: its limitations as used in higher education evaluation. Int J Assess Tools Educ.

[25] Haji F, Morin MP, Parker K (2013). Rethinking programme evaluation in health professions education: beyond “did it work?”. Med Educ.

[26] Allen LM, Hay M, Palermo C (2022). Evaluation in health professions education—is measuring outcomes enough?. Med Educ.

[27] Yardley S, Dornan T (2012). Kirkpatrick’s levels and education “evidence”. Med Educ.

[28] Newman JH (2008). The Idea of a University Defined and Illustrated Project.

[29] Sandars J, Patel RS, Goh PS, Kokatailo PK, Lafferty N (2015). The importance of educational theories for facilitating learning when using technology in medical education. Med Teach.

[30] Shalong W, Yi Z, Bin Z (2025). Enhancing self-directed learning with custom GPT AI facilitation among medical students: a randomized controlled trial. Med Teach.

[31] Clark J, Glasziou P, Del Mar C, Bannach-Brown A, Stehlik P, Scott AM (2020). A full systematic review was completed in 2 weeks using automation tools: a case study. J Clin Epidemiol.

[32] Charmaz K (2014). Constructing Grounded Theory.

[33] Braun V, Clarke V (2019). Reflecting on reflexive thematic analysis. Qual Res Sport Exerc Health.

[34] Jowsey T, Braun V, Clarke V, Lupton D, Fine M (2025). We reject the use of generative artificial intelligence for reflexive qualitative research. Qual Inq.

[35] Roberts J, Baker M, Andrew J (2024). Artificial intelligence and qualitative research: the promise and perils of large language model (LLM) ‘assistance’. Crit Perspect Account.

[36] Hitch D (2024). Artificial intelligence augmented qualitative analysis: the way of the future?. Qual Health Res.

[37] Bland T (2025). Enhancing medical student engagement through cinematic clinical narratives: multimodal generative AI-based mixed methods study. JMIR Med Educ.

[38] Worthley B, Guo M, Sheneman L, Bland T (2025). Antiparasitic pharmacology goes to the movies: leveraging generative AI to create educational short films. AI.

[39] Thesen T, O’Brien WN, Stone S, Pinto-Powell R (2025). Generative AI as the first patient: practice, feedback, and confidence. Med Sci Educ.

[40] Holderried F, Stegemann-Philipps C, Herschbach L (2024). A generative pretrained transformer (GPT)–powered chatbot as a simulated patient to practice history taking: prospective, mixed methods study. JMIR Med Educ.

[41] Alomar Z, Guo M, Bland T (2025). AI-generated mnemonic images improve long-term retention of coronary artery occlusions in STEMI: a comparative study. Technologies (Basel).

[42] Amano I, Obi-Nagata K, Ninomiya A, Fujiwara Y, Koibuchi N (2025). Use and evaluation of generative artificial intelligence by medical students in Japan. JMA J.

[43] Borg A, Jobs B, Huss V (2024). Enhancing clinical reasoning skills for medical students: a qualitative comparison of LLM-powered social robotic versus computer-based virtual patients within rheumatology. Rheumatol Int.

[44] Ebihara H, Kasai H, Shimizu I (2025). Development of a clinical clerkship mentor using generative AI and evaluation of its effectiveness in a medical student trial compared to student mentors: 2-part comparative study. JMIR Med Educ.

[45] Laverde N, Grévisse C, Jaramillo S, Manrique R (2025). Integrating large language model-based agents into a virtual patient chatbot for clinical anamnesis training. Comput Struct Biotechnol J.

[46] Liu Y, Shi C, Wu L (2025). Development and validation of a large language model–based system for medical history-taking training: prospective multicase study on evaluation stability, human-AI consistency, and transparency. JMIR Med Educ.

[47] Peralta Ramirez AA, Trujillo López S, Navarro Armendariz GA, De la Torre Othón SA, Sierra Cervantes MR, Medina Aguirre JA (2025). Clinical simulation with ChatGPT: a revolution in medical education?. J CME.

[48] Tekin A, Karamus NF, Çolak T (2025). Anatomy exam model for the circulatory and respiratory systems using GPT-4: a medical school study. Surg Radiol Anat.

[49] Borg A, Georg C, Jobs B (2025). Virtual patient simulations using social robotics combined with large language models for clinical reasoning training in medical education: mixed methods study. J Med Internet Res.

[50] Dupont V, Guerrot D, Bouazzi L (2025). Evaluating podcasts as a tool for OSCE training: a randomized trial using generative AI-powered simulation. BMC Med Educ.

[51] Hassoulas A, Crawford O, Hemrom S (2025). A pilot study investigating the efficacy of technology enhanced case based learning (CBL) in small group teaching. Sci Rep.

[52] Li L, Zhang W, Zhang K (2025). The role of generative AI tools in case-based learning and teaching evaluation of medical biochemistry. BMC Med Educ.

[53] Mashburn P, Weuthen FA, Otte N (2025). Gender differences in the use of ChatGPT as generative artificial intelligence for clinical research and decision-making in occupational medicine. Healthcare (Basel).

[54] Suárez-García RX, Chavez-Castañeda Q, Orrico-Pérez R (2025). DIALOGUE: a generative AI-based pre–post simulation study to enhance diagnostic communication in medical students through virtual type 2 diabetes scenarios. Eur J Investig Health Psychol Educ.

[55] Wang Z, Fan TT, Li ML, Zhu NJ, Wang XC (2025). Feasibility study of using GPT for history-taking training in medical education: a randomized clinical trial. BMC Med Educ.

[56] Weuthen FA, Otte N, Krabbe H, Kraus T, Krabbe J (2025). Comparison of ChatGPT and internet research for clinical research and decision-making in occupational medicine: randomized controlled trial. JMIR Form Res.

[57] Yamamoto A, Koda M, Ogawa H (2024). Enhancing medical interview skills through AI-simulated patient interactions: nonrandomized controlled trial. JMIR Med Educ.

[58] Brügge E, Ricchizzi S, Arenbeck M (2024). Large language models improve clinical decision making of medical students through patient simulation and structured feedback: a randomized controlled trial. BMC Med Educ.

[59] Gan W, Ouyang J, Li H (2024). Integrating ChatGPT in orthopedic education for medical undergraduates: randomized controlled trial. J Med Internet Res.

[60] Montagna M, Chiabrando F, De Lorenzo R, Rovere Querini P, Medical Students (2025). Impact of clinical decision support systems on medical students’ case-solving performance: comparison study with a focus group. JMIR Med Educ.

[61] Kalam KA, Masoud FD, Muntaser A, Ranga R, Geng X, Goyal M (2025). ChatGPT as a learning tool for medical students: results from a randomized controlled trial. Cureus.

[62] Skryd A, Lawrence K (2024). ChatGPT as a tool for medical education and clinical decision-making on the wards: case study. JMIR Form Res.

[63] Wu Q, Wang Y, Lu L, Chen Y, Long H, Wang J (2022). Virtual simulation in undergraduate medical education: a scoping review of recent practice. Front Med.

[64] Roossien L, van Klaveren LM, de Vos R, Dolmans DHJM, Boerboom TBB (2025). Team-based learning (TBL) in health professions education: a systematic review on its conceptual elements and outcomes. Med Teach.

[65] Curran VR, Fleet L (2005). A review of evaluation outcomes of web-based continuing medical education. Med Educ.

[66] Kulaksız T, Steinbacher J, Kalz M (2023). Technology-enhanced learning in the education of oncology medical professionals: a systematic literature review. J Canc Educ.

[67] Delungahawatta T, Dunne SS, Hyde S (2022). Advances in e-learning in undergraduate clinical medicine: a systematic review. BMC Med Educ.

[68] Ludmerer KM (2005). Time to Heal: American Medical Education from the Turn of the Century to the Era of Managed Care.

[69] Aster A, Laupichler MC, Rockwell-Kollmann T, Masala G, Bala E, Raupach T (2025). ChatGPT and other large language models in medical education—scoping literature review. Med Sci Educ.

[70] Gordon M, Daniel M, Ajiboye A (2024). A scoping review of artificial intelligence in medical education: BEME Guide No. 84. Med Teach.

[71] Göttgens I, Oertelt-Prigione S (2021). The application of human-centered design approaches in health research and innovation: a narrative review of current practices. JMIR Mhealth Uhealth.

[72] Mummah SA, Robinson TN, King AC, Gardner CD, Sutton S (2016). IDEAS (Integrate, Design, Assess, and Share): a framework and toolkit of strategies for the development of more effective digital interventions to change health behavior. J Med Internet Res.

[73] Gasson S (2003). Human-centered vs. user-centered approaches to information system design. J Inf Technol Theory Appl.

