# Supplementary material for: The Forgotten Student—Generative AI Use in Medical Education and the Technology-Integrated Educational Design (TIED) Road Map: Scoping Review
Source: J Med Internet Res. 2026 Oct 15;28:e96099. doi: 10.2196/96099 (PMC13671686; doi:10.2196/96099)
Supplement: Supplementary file 1 [file jmir-v28-e96099-s001.docx]

**Search Strategy:**

The Population–Concept–Context^[1]^:

- Population: undergraduate medical students;
- Concept: use of generative AI (GenAI) in medical education
- Context: education-related empirical research (teaching/learning intervention).

Databases:

- Ovid (Medline, Embase): The MEDLINE and Embase strategies were run simultaneously as a multi-file search in Ovid.
- PubMed
- Web of Science
- Scopus

Dates of search: 22–24 September 2025

Search string used in Pubmed on titles and abstracts (and translated to other databases searches)

("GenAI"[Title/Abstract] OR "generative AI"[Title/Abstract] OR "large language model"[Title/Abstract] OR "gpt"[Title/Abstract] OR "deepseek"[Title/Abstract] OR "LLM"[Title/Abstract]) AND ("medical student"[Title/Abstract] OR "medical education"[Title/Abstract])

Filters: no language, date or publication-type restrictions.

No gray literature was searched

Software:

- Software used for deduplication: TERA tools
- Software used for screening: TERA tools
- Software used for full text screening and data extraction: MsExcel

Search validation:

- Review of existing approaches (see: Kiyak et al 2026, Fiegerlova et al 2025).
- Pilot search analyzed by the team collectively to determine recall and precision.

Review stages

- The scoping review was undertaken in accordance with Arksey and O’Malley framework and included formulating the research question, identifying relevant evidence, evidence search, selecting eligible evidence, charting the data, data synthesis.

1. Peters, M. D. J., Marnie, C., Tricco, A. C., Pollock, D., Munn, Z., Alexander, L., McInerney, P., Godfrey, C. M., & Khalil, H. (2020). Updated methodological guidance for the conduct of scoping reviews. JBI Evidence Synthesis, 18(10), 2119–2126. <https://doi.org/10.11124/JBIES-20-00167>
