# Supplementary material for: The Forgotten Student—Generative AI Use in Medical Education and the Technology-Integrated Educational Design (TIED) Road Map: Scoping Review
Source: J Med Internet Res. 2026 Oct 15;28:e96099. doi: 10.2196/96099 (PMC13671686; doi:10.2196/96099)
Supplement: Supplementary file 2 [file jmir-v28-e96099-s002.docx]

| **Study Characteristics** | | | | | | | **Kirkpatrick's level** | | | |  |
| --- | --- | --- | --- | --- | --- | --- | --- | --- | --- | --- | --- |
| **Reference** | **Aim** | **Study design** ^[1]^ | **Use of GenAI** | **GenAI tool** | **Results** | **Data collection**^[2]^ | **Level 1**  **Reaction** | **Level 2**  **Learning** | **Level 3**  **Behaviour (behavior enacted consistently and continuously)** | **Level 4**  **System (Clinical/Patient outcomes or Pedagogical grounding):** | **Level “how it works”** |
| Amano et al. [1] | To investigate how generative AI is perceived and utilized among medical students. | QN | knowledge/decision support | GPT | Low use of GenAI before the study. Rather negative viewpoint on the use of GenAI for medical purposes but willingness to using it in the future beyond medical contexts. | S | ✓^[3]^ | ✕ | ✕ | ✕ | ✕ |
| Bland 2025 [2] | To evaluate the effectiveness of genAI-enhanced cinematic clinical narrative in increasing student engagement, interest, and knowledge retention. | QN* | educational content generation | GPT+other | Preference for the genAI-enhanced content over traditional teaching methods. High average scores for triggered situational interest, maintained interest, maintained-feeling interest, and maintained-value interest. | S, T, VQ | ✓ | ✕ | ✕ | ✕  Implicit: Cinematic  Clinical Narratives but vaguely grounded in learning theories and not discussed; more through study design: situational interest theory not discussed in detail in the discussion. | ✕ |
| Borg et al. 2024 [3] | To explore the potential added value of a social robotic VP platform, enriched with an LLM. | QL | simulated patient | GPT | More authentic and interactive learning experience compared to the conventional computer-based platforms. Three main themes were identified: authenticity, VP application, and strengths and limitations. | I | ✓ | ✕ | ✕ | ✕  Implicit: simulation with GenAI used but not grounded learning theories and not connected with study design and discussion. | ✕ |
| Ebihara et al.  [4] | To evaluate the effectiveness of AI-based system in assisting with clinical queries and alleviating psychological stress. | QN | knowledge/decision support | GPT | Positive perceptions: moderately useful, with positive evaluations for clarity and emotional support. Aspects related to feedback guidance and anxiety reduction received more neutral ratings. Students primarily consulted the tool regarding learning workload and communication difficulties; few students used it to address emotional stress–related issues. | CL, S, VQ | ✓ | ✕ | ✕ | ✕  Implicit: technology acceptance model used as a framework, no grounding in learning theory. | ✕ |
| Holderried et al. [5] | To investigate the potential of GenAI as a simulated patient. | QN* | simulated patient | GPT | Overall positive user experience. | CL, VQ | ✓ | ✕ | ✕  History-taking skills - not tested in clinical settings nor as a sustained clinical behavior. | ✕  Implicit: simulation with GenAI used but not grounded learning theories. | ✕ |
| Laverde et al. [6] | To explore the potential of LLM-based GenAgent as Virtual Patients for clinical history-taking training. | QN | simulated patient | Custom | Overall positive user experience. | CL, S, VQ | ✓ | ✕ | ✕  History-taking tool: not tested in clinical settings nor as one-off or sustained clinical behavior. | ✕  Implicit: simulation with GenAI used but not grounded learning theories. | ✕ |
| Liu et al. [7] | To develop and validate the AI-Powered Medical History-Taking Training and Evaluation System. | QN | simulated patient (+ feedback) | Deepseek | Helpful. Expression of a desire to use it again in the future. | S | ✓ | ✕ | ✕  History-taking tool: not tested in clinical settings nor as one-off or sustained clinical behavior. | ✕  Implicit: simulation with GenAI used but not grounded in learning theories. | ✕ |
| Peralta Ramirez et al. [8] | To evaluate medical students’ perceptions of GenAI-created clinical simulation scenarios. | QN | simulated patient | GPT | High satisfaction (clarity, appropriateness, usefulness). | S | ✓ | ✕ | ✕ | ✕  Implicit: simulation with GenAI used but not grounded in learning theories determining study design and discussion. | ✕ |
| Tekin et al. [9] | To evaluate the effectiveness of multiple-choice questions generated by GenAI. | QN | educational content generation | GPT | Students achieved good results on the test. GenAI output found mostly clear. High interest in retaking the assessment exam. | S, T | ✓ | ✕ | ✕ | ✕ | ✕ |
| Thesen et al. [10] | To examine experiences and attitudes toward GenAI patient actor in clinical education. | QN* | simulated patient | GPT+other | Positive experiences, appreciation for the AI approaches’ flexibility, low-pressure environment, and real-time feedback. Students identified limitations regarding the authenticity of AI interactions compared to human encounters. | S | ✓ | ✕ | ✕ | ✕  Implicit: simulation with GenAI used, vaguely described in the context of learning theories (tailored instruction, situated learning theory, deliberate practice theory, self-determination theory) not connected with study design and discussion. | ✕ |
| Borg et al. 2025 [11] | To explore the potential of a social robotic VP platform combined with an LLM compared to a conventional computer-based VP modality. | M | simulated patient | GPT+other | High perceptions of the tool (authenticity, beneficial learning effect).  Qualitative analysis revealed the following themes: realism and immersion, skill acquisition and development, procedural limitations and potential for improvement. | I, VQ | ✓ | ✓ | ✕ | ✕  Implicit: simulation with GenAI used but vaguely grounded learning theories. | ✕ |
| Dupont et al. [12] | To evaluate the effectiveness of OSCE training podcast as a supplementary tool for enhancing the performance of undergraduate medical students, assessed through a generative AI-powered virtual OSCE platform. | QN | simulated patient | GPT+other | After the intervention, the podcast group demonstrated a significantly higher OSCE score compared to the control group with a greater improvement in OSCE scores. | S, T | ✓ | ✓ | ✕  Communication skills - not tested in clinical settings nor as a sustained clinical behavior. | ✕ | ✕ |
| Hassoulas et al. [13] | To investigate the feasibility of delivering technology enhanced learning (TEL) and case-based learning (CBL). | QN | simulated patient | GPT+other | Better performance in formative assessments. | S, T | ✓ | ✓ | ✕  Clinical decision tool - not tested in clinical settings nor for one-off or sustained clinical behavior. | ✕  Implicit: simulation with GenAI used but not grounded in learning theories (technology-enhanced learning is a research context not a learning theory) and not connected with study design and discussion. | ✕ |
| Li et al. [14] | To explore the potential of GenAI tools as teaching assistants in Case-Based Learning settings. | QN | knowledge/decision support | Kimi chat | Faster completion rates and higher exam scores. High students rating of GenAI for basic knowledge acquisition, low on complex clinical reasoning and innovative thinking. Key concerns included reduced interaction between teachers and students and standardized AI outputs that led to homogenized learning. Students’ acceptance increased significantly after the trial. | CL, S, T | ✓ | ✓ | ✕ | ✕ | ✕ |
| Mashburn et al. [15] | To explore gender-specific differences in the use of ChatGPT by medical students and physicians while solving occupational lung disease cases. | QN | knowledge/decision support | GPT | Women showed greater knowledge improvement and higher self-rated competence. No significant gender differences emerged in diagnostic accuracy, reporting procedures, or satisfaction with the tool. | CL, S | ✓ | ✓ | ✕ | ✕ | ✕ |
| Suárez-García et al. [16] | To evaluate the effectiveness of a GenAI-driven simulation for training of diagnostic communication skills in medical students. | QN | simulated patient (+ feedback) | GPT | Increased performance as well as the proportion of high-performing students. Lower baseline empathy and higher digital self-efficacy independently predicted greater improvement. | CL, S, T, VQ | ✓ | ✓ | ✕  Communication skills gained through simulation - not tested in clinical settings nor as a sustained clinical behavior. One-off evaluation of diagnostic communication domains. | ✕  Implicit: simulation with GenAI used, vaguely described in the context of learning theories. | ✕  Describes changes in scores in different dimensions of diagnostic communication but does not explain how GenAI impacted them. |
| Wang et al. [17] | To evaluate the effectiveness of GenAI simulated patient system in improving medical students’ history-taking skills. | QN | simulated patient | GPT | GPT-simulation group showed significantly higher post-training scores in the structured clinical examination compared to the control group. Significantly higher scores, enthusiasm for learning, self-directed learning motivation, communication feedback abilities, and satisfaction (ease of use, likelihood of recommending the training). | S, T | ✓ | ✓ | ✕  History-taking skills - not tested in clinical settings nor as a sustained clinical behavior. | ✕  Implicit: simulation with GenAI used but not grounded learning theories. | ✕ |
| Weuthen et al. [18] | To assess the potential suitability of a generative LLM as a support tool for medical research and clinical decisions. | QN | knowledge/decision support | GPT | Better performance in specific research as well as clinical decisions. Increase in self-assessment with regard to specialist  knowledge. | S, T | ✓ | ✓ | ✕ | ✕ | ✕ |
| Worthley et al. [19] | To evaluate the impact of a short film on learning outcomes and student engagement. | QN* | educational content generation | GPT+other | Higher score on exam questions in comparison to control, improved engagement and interest; preference towards GenAI outputs in comparison to other formats. | S, T, VQ | ✓ | ✓ | ✕ | ✕  Implicit: Cinematic  Clinical Narratives but vaguely grounded in learning theories; more through study design: situational interest theory which is not mentioned but its instrument is used. | ✕ |
| Yamamoto et al. [20] | To investigate whether medical students’ interview skills could be improved by engaging with AI-simulated patients, including the provision of feedback. | QN | simulated patient (with feedback) | GPT+other | Significantly higher scores in medical interviews. | CL, S, T, VQ | ✓ | ✓ | ✕  Medical interviewing skills - not tested in clinical settings nor as a sustained clinical behavior. (one-off testing). | ✕  Implicit: simulation with GenAI used but not grounded in learning theories determining study design and discussion. | ✕ |
| Gan et al. [21] | To evaluate GenAI accuracy in answering orthopedics-related MCQs and assess its short-term effects as a learning aid. | QN | knowledge/decision support | GPT | Better test and exam performance after the research team intervened in the learning style of the students. | T | ✕ | ✓ | ✕ | ✕ |  |
| Brügge et al. [22] | To explore the potential of using LLM to simulate patient-doctor interactions and provide structured feedback. | QN | simulated patient (+feedback) | GPT | Better performance based on clinical reasoning indicator. | VQ | ✕ | ✓ | ✕  Clinical decision- making not tested in clinical settings nor as one-off nor sustained clinical behavior. | ✕  Implicit: simulation with GenAI used but vaguely grounded in learning theories (feedback) and not connected with study design and discussion. | ✕  Describes changes in scores in different dimensions of clinical decision- making but does not explain how GenAI impacted them. |
| Montagna et al. [23] | To explore how medical students perform in an evaluated clinical case through the use of different clinical decision support systems tools. | QN | knowledge/decision support | GPT | Faster answering time, but the clinical practice guidance group performed best in nearly all domains and maintained almost perfect alignment between its completeness and accuracy. | CL, T | ✕ | ✓ | ✕ | ✕ | ✕ |
| Alomar et al. [24] | To explore using generative AI to create mnemonic-based images to enhance human learning and retention of medical images. | QN | educational content generation | GPT+other | Stable scores on the relevant exam question over time, significantly higher situational interest. | S, T, VQ | ✓ | ✓ | ✕ | ✓  Explicit: mnemonic techniques grounded in learning theories but GenAI integration is not theorized. | ✕  The discussion touches upon the “how” behind mnemonic techniques but not on how GenAI contributes to the results of the intervention and why it matters. |
| Shalong et al. [25] | To assess the impact of GenAI tool designed to support self-directed learning among medical students. | M | knowledge/decision support | GPT | Marginally higher median Self-Directed Learning Scale score, which further improved by 12 weeks and was sustained at the 14-week follow-up. Notable improvements in the Cornell Critical Thinking Test Score at 12 weeks, which persisted into the 14-week follow-up. | S, VQ | ✓ | ✓ | ✕ | ✓  Explicit: self-directed learning (Self-Directed Learning Scale and the  Cornell Critical Thinking Test scales). | ✕ |
| Kalam et al. [26] | To evaluate short‑term academic performance and retention; to collect and analyze student perceptions; to interpret the results within the framework of Cognitive Load Theory; and collect and analyze student perceptions. | QN | knowledge/decision support | GPT | Initial quiz scores, however, retention scores one week later showed no significant differences among the groups. | S, T | ✓ | ✓ | ✕ | ✓  Explicit: GenAI integration grounded in Cognitive Load Theory, connected with the study design and discussion. | ✓  Discusses how GPT reduces extraneous cognitive load for short-term gains. |
| Skryd et al. [27] | To identify and evaluate potential use cases and limitations of LLM in real-time ward-based educational contexts. | QL | knowledge/decision support | GPT | Themes: discrete medical knowledge inquiries, building differential diagnoses and engaging dual process thinking, challenging medical axioms, using cognitive aids to support acute care decision-making, and facilitating conversations with subspecialties and other. | CL, I | ✕ | ✕ | ✕ | ✕ | ✓  As described in themes. |

References for the appendix 2:

1.	Amano I, Obi-Nagata K, Ninomiya A, Fujiwara Y, Koibuchi N. Use and Evaluation of Generative Artificial Intelligence by Medical Students in Japan. JMA J Japan; 2025 July 15;8(3):730–735. PMID:40786463

2.	Bland T. Enhancing Medical Student Engagement Through Cinematic Clinical Narratives: Multimodal Generative AI-Based Mixed Methods Study. JMIR medical education 2025;11:e63865. doi: https://dx.doi.org/10.2196/63865

3.	Borg A, Jobs B, Huss V, Gentline C, Espinosa F, Ruiz M, Edelbring S, Georg C, Skantze G, Parodis. Enhancing clinical reasoning skills for medical students: a qualitative comparison of LLM-powered social robotic versus computer-based virtual patients within rheumatology. Rheumatology International 2024;44(12):3041–3051. doi: https://dx.doi.org/10.1007/s00296-024-05731-0

4.	Ebihara H, Kasai H, Shimizu I, Shikino K, Tajima H, Kimura Y, Ito S. Development of a Clinical Clerkship Mentor Using Generative AI and Evaluation of Its Effectiveness in a Medical Student Trial Compared to Student Mentors: 2-Part Comparative Study. JMIR Med Educ Canada; 2025 Sept 4;11:e76702. PMID:40907969

5.	Holderried F, Stegemann-Philipps C, Herschbach L, Moldt J-A, Nevins A, Griewatz J, Holderried M, Herrmann-Werner A, Festl-Wietek T, Mahling M. A Generative Pretrained Transformer (GPT)-Powered Chatbot as a Simulated Patient to Practice History Taking: Prospective, Mixed Methods Study. JMIR Med Educ Canada; 2024 Jan 16;10:e53961. PMID:38227363

6.	Laverde N, Grévisse C, Jaramillo S, Manrique R. Integrating large language model-based agents into a virtual patient chatbot for clinical anamnesis training. Comput Struct Biotechnol J Netherlands; 2025;27:2481–2491. PMID:40547455

7.	Liu Y, Shi C, Wu L, Lin X, Chen X, Zhu Y, Tan H, Zhang W. Development and Validation of a Large Language Model–Based System for Medical History-Taking Training: Prospective Multicase Study on Evaluation Stability, Human-AI Consistency, and Transparency. JMIR Medical Education JMIR Publications Inc., Toronto, Canada; 2025 Aug 29;11(1):e73419. doi: 10.2196/73419

8.	Peralta Ramirez A, Trujillo López S, Navarro Armendariz GA G, De la Torre Othón S, Sierra Cervantes MR M, Medina Aguirre J. Clinical Simulation with ChatGpt: A Revolution in Medical Education? J CME 2025;14(1):2525615. doi: 10.1080/28338073.2025.2525615

9.	Tekin A, Karamus NF, Çolak T. YES Anatomy exam model for the circulatory and respiratory systems using GPT-4: a medical school study. Surg Radiol Anat Germany; 2025 June 10;47(1):158. PMID:40495075

10.	Thesen T, O’Brien WN, Stone S, Pinto-Powell R. Generative AI as the First Patient: Practice, Feedback, and Confidence. Medical Science Educator 2025; doi: 10.1007/s40670-025-02473-x

11.	Borg A, Georg C, Jobs B, Huss V, Waldenlind K, Ruiz M, Edelbring S, Skantze G, Parodis I. Virtual Patient Simulations Using Social Robotics Combined With Large Language Models for Clinical Reasoning Training in Medical Education: Mixed Methods Study. Journal of Medical Internet Research 2025;27:e63312. doi: https://dx.doi.org/10.2196/63312

12.	Dupont V, Guerrot D, Bouazzi L, Figueres L, Pers Y-M, Guenou E, Barbe C, Yauy K. Evaluating podcasts as a tool for OSCE training: a randomized trial using generative AI-powered simulation. BMC medical education 2025;25(1):429. doi: https://dx.doi.org/10.1186/s12909-025-06675-0

13.	Hassoulas A, Crawford O, Hemrom S, de Almeida A, Coffey MJ, Hodgson M, Leveridge B, Karwa D, Lethbridge A, Williams H, Voisey A, Reed K, Patel S, Hart K, Shaw H. A pilot study investigating the efficacy of technology enhanced case based learning (CBL) in small group teaching. Sci rep England; 2025;15(1):15604. doi: 10.1038/s41598-025-99764-5

14.	Li L, Zhang W, Zhang K, Yang Y, Wang L, Zuo L, Sun Y, Peng Q. The role of generative AI tools in case-based learning and teaching evaluation of medical biochemistry. BMC Med Educ England; 2025 Aug 22;25(1):1185. PMID:40847414

15.	Mashburn P, Weuthen FA, Otte N, Krabbe H, Fernandez GM, Kraus T, Krabbe J. Gender Differences in the Use of ChatGPT as Generative Artificial Intelligence for Clinical Research and Decision-Making in Occupational Medicine. Healthcare (Basel) 2025 June 11;13(12). doi: 10.3390/healthcare13121394

16.	Suárez-García RX, Chavez-Castañeda Q, Orrico-Pérez R, Valencia-Marin S, Castañeda-Ramírez AE, Quiñones-Lara E, Ramos-Cortés CA, Gaytán-Gómez AM, Cortés-Rodríguez J, Jarquín-Ramírez J, Aguilar-Marchand NG, Valdés-Hernández G, Campos-Martínez TE, Vilches-Flores A, Leon-Cabrera S, Méndez-Cruz AR, Jay-Jímenez BO, Saldívar-Cerón HI. DIALOGUE: A Generative AI-Based Pre-Post Simulation Study to Enhance Diagnostic Communication in Medical Students Through Virtual Type 2 Diabetes Scenarios. Eur J Investig Health Psychol Educ Switzerland; 2025 Aug 7;15(8). PMID:40863274

17.	Wang Z, Fan T-T, Li M-L, Zhu N-J, Wang X-C. Feasibility study of using GPT for history-taking training in medical education: a randomized clinical trial. BMC Med Educ England; 2025 July 10;25(1):1030. PMID:40640776

18.	Weuthen FA, Otte N, Krabbe H, Kraus T, Krabbe J. Comparison of ChatGPT and Internet Research for Clinical Research and Decision-Making in Occupational Medicine: Randomized Controlled Trial. JMIR Form Res 2025 May 20;9:e63857. PMID:40393042

19.	Worthley B, Guo M, Sheneman L, Bland T. Antiparasitic Pharmacology Goes to the Movies: Leveraging Generative AI to Create Educational Short Films. AI (Switzerland) 2025;6(3). doi: 10.3390/ai6030060

20.	Yamamoto A, Koda M, Ogawa H, Miyoshi T, Maeda Y, Otsuka F, Ino H. Enhancing Medical Interview Skills Through AI-Simulated Patient Interactions: Nonrandomized Controlled Trial. JMIR Med Educ Canada; 2024 Sept 23;10:e58753. PMID:39312284

21.	Brügge E, Ricchizzi S, Arenbeck M, Keller MN, Schur L, Stummer W, Holling M, Lu MH, Darici D. Large language models improve clinical decision making of medical students through patient simulation and structured feedback: a randomized controlled trial. BMC Medical Education 2024;24(1). doi: 10.1186/s12909-024-06399-7

22.	Gan W, Ouyang J, Li H, Xue Z, Zhang Y, Dong Q, Huang J, Zheng X, Zhang Y. Integrating ChatGPT in Orthopedic Education for Medical Undergraduates: Randomized Controlled Trial. J Med Internet Res Canada; 2024 Aug 20;26:e57037. PMID:39163598

23.	Montagna M, Chiabrando F, De Lorenzo R, Rovere Querini P. Impact of Clinical Decision Support Systems on Medical Students? Case-Solving Performance: Comparison Study with a Focus Group. JMIR Med Educ 2025 Mar 18;11:e55709. doi: 10.2196/55709

24.	Alomar Z, Guo M, Bland T. AI-Generated Mnemonic Images Improve Long-Term Retention of Coronary Artery Occlusions in STEMI: A Comparative Study. Technologies 2025;13(6). doi: 10.3390/technologies13060217

25.	Shalong W, Yi Z, Bin Z, Ganglei L, Jinyu Z, Yanwen Z, Zequn Z, Lianwen Y, Feng R. Enhancing self-directed learning with custom GPT AI facilitation among medical students: A randomized controlled trial. Med Teach England; 2025 July;47(7):1126–1133. PMID:39425996

26.	Kalam KA, Masoud FD, Muntaser A, Ranga R, Geng X, Goyal M. ChatGPT as a Learning Tool for Medical Students: Results From a Randomized Controlled Trial. Cureus United States; 2025 June;17(6):e85767. PMID:40656250

27.	Skryd A, Lawrence K. ChatGPT as a Tool for Medical Education and Clinical Decision-Making on the Wards: Case Study. JMIR Form Res 2024 May 8;8:e51346. doi: 10.2196/51346

1. QL – Quantitative, QN – Quantitative, QN* - classified as quantitative because of automated AI coding, M – Mixed
2. CL - conversation logs/analytics, I - Interview, S – survey, incl. with open-ended questions, T - exam, test, OSCE, VQ - validated questionnaire
3. ✓ indicates the characteristic was present; ✕ indicates it was absent.
