# Supplementary material for: The Forgotten Student—Generative AI Use in Medical Education and the Technology-Integrated Educational Design (TIED) Road Map: Scoping Review
Source: J Med Internet Res. 2026 Oct 15;28:e96099. doi: 10.2196/96099 (PMC13671686; doi:10.2196/96099)
Supplement: Supplementary file 3 [file jmir-v28-e96099-s003.docx]

**Level 0: level 0.1 and 0.2**

Duplicates, n = 3

Excluded based on tightened criteria, n = 221, including

Level 0.1 – no intervention n = 111

Level 0.2 – performance, n = 110

Records considered

for tighter eligibility criteria

n = 269

Reports excluded:

- Not medical education, conference papers, unretrievable (10)
- Based on tightened criteria (8 for level 0.1)

n = 18

**Included**

Studies sought for retrieval and full-text screening

n = 45

8 records added to level 0.1: 111 + 8 = 119

Level 0.1 – no intervention n = 119

Level 0.2 – GenAI performance, n = 110

n = 229

Studies included in the review

n = 27

**Level 0.2: GenAI performance (n=110)**

| LP | Year | Authors | Title |
| --- | --- | --- | --- |
| 1 | 2023 | Kilic M. E, Kilic M. E, et al. | AI in Medical Education: A Comparative Analysis of GPT-4 and GPT-3.5 on Turkish Medical Specialization Exam Performance |
| 2 | 2023 | Roos, J., Kasapovic, A., Kaczmarczyk, R., et al. | Artificial Intelligence in Medical Education: Comparative Analysis of ChatGPT, Bing, and Medical Students in Germany |
| 3 | 2023 | Banerjee, A., Ahmad, A., Goyal, K., et al. | Assessing the Efficacy of ChatGPT in Solving Questions Based on the Core Concepts in Physiology |
| 4 | 2023 | Meaney C, Huang R. S, Punnett A, et al. | Comparing the Performance of ChatGPT and GPT-4 versus a Cohort of Medical Students on an Official University of Toronto Undergraduate Medical Education Progress Test Technical Report |
| 5 | 2023 | Ignjatović A, Stevanović L, Stevanović L, et al. | Efficacy and limitations of ChatGPT as a biostatistical problem-solving tool in medical education in Serbia: a descriptive study |
| 6 | 2023 | Lai, U. H., Wu, K. S., Kan, J. K. C., et al. | Evaluating the performance of ChatGPT-4 on the United Kingdom Medical Licensing Assessment |
| 7 | 2023 | Rosoł, M., Gąsior, J. S., Młyńczak, M., et al. | Evaluation of the performance of GPT-3.5 and GPT-4 on the Polish Medical Final Examination |
| 8 | 2023 | Jang, D., Yun, T. R., Kim, C. E., et al. | GPT-4 can pass the Korean National Licensing Examination for Korean Medicine Doctors |
| 9 | 2023 | Gilson, A., Safranek, C. W., Chartash, D., et al. | How Does ChatGPT Perform on the United States Medical Licensing Examination (USMLE)? The Implications of Large Language Models for Medical Education and Knowledge Assessment |
| 10 | 2023 | Ghosh, A., Maini Jindal N, Sett, A., et al. | Is ChatGPT's Knowledge and Interpretative Ability Comparable to First Professional MBBS (Bachelor of Medicine, Bachelor of Surgery) Students of India in Taking a Medical Biochemistry Examination? |
| 11 | 2023 | Flores-Cohaila, J. A., García-Vicente A, Taype-Rondan, A., et al. | Performance of ChatGPT on the Peruvian National Licensing Medical Examination: Cross-Sectional Study |
| 12 | 2023 | Borchert, R. J., Hickman, C. R., Sadler, T. J., et al. | Performance of ChatGPT on the Situational Judgement Test-A Professional Dilemmas-Based Examination for Doctors in the United Kingdom |
| 13 | 2023 | Giannos, P., Delardas, O., Delardas, O., et al. | Performance of ChatGPT on UK Standardized Admission Tests: Insights from the BMAT, TMUA, LNAT, and TSA Examinations |
| 14 | 2023 | Kung, T. H., Cheatham, M., Maningo, J., et al. | Performance of ChatGPT on USMLE: Potential for AI-assisted medical education using large language models |
| 15 | 2023 | Torres-Zegarra, B. C., Rios-Garcia, W., Flores-Cohaila, J. A., et al. | Performance of ChatGPT, Bard, Claude, and Bing on the Peruvian National Licensing Medical Examination: a cross-sectional study |
| 16 | 2023 | Takagi, S., Watari, T., Sakaguchi, K., et al. | Performance of GPT-3.5 and GPT-4 on the Japanese Medical Licensing Examination: Comparison Study |
| 17 | 2023 | Dhanvijay, A. K. D., Pinjar, M. J., Mondal, H., et al. | Performance of Large Language Models (ChatGPT, Bing Search, and Google Bard) in Solving Case Vignettes in Physiology |
| 18 | 2023 | Husain, S. S., Ansari, Z., Mujahid, R., et al. | To Evaluate the Efficiency of ChatGPT in Medical Education: An Analysis of MCQ-Based Learning and Assessment |
| 19 | 2024 | Alessandri Bonetti, M., Liu, H. Y., Egro, F. M., et al. | A Comparative Analysis of ChatGPT, ChatGPT-4, and Google Bard Performances at the Advanced Burn Life Support Exam |
| 20 | 2024 | Mondal, H., De, R., Juhi, A., et al. | A large language model in solving primary healthcare issues: A potential implication for remote healthcare and medical education |
| 21 | 2024 | Penny, P., Bane, R., Riddle, V., et al. | Advancements in AI Medical Education: Assessing ChatGPT's Performance on USMLE-Style Questions Across Topics and Difficulty Levels |
| 22 | 2024 | Cherif, H., Moussa, C., Dhahri, B., et al. | Appraisal of ChatGPT's Aptitude for Medical Education: Comparative Analysis With Third-Year Medical Students in a Pulmonology Examination |
| 23 | 2024 | Herrmann-Werner, A., Festl-Wietek, T., Mahling, M., et al. | Assessing ChatGPT's Mastery of Bloom's Taxonomy Using Psychosomatic Medicine Exam Questions: Mixed-Methods Study |
| 24 | 2024 | Chen, J., Cadiente, A., Pilkington, B., et al. | Assessing the performance of ChatGPT in bioethics: a large language model's moral compass in medicine |
| 25 | 2024 | Ranjan, J., Ahmad, A., Kumar, A., et al. | Assessment of Artificial Intelligence Platforms With Regard to Medical Microbiology Knowledge: An Analysis of ChatGPT and Gemini |
| 26 | 2024 | Roy, S., Khatua, A., Ganguly, N., et al. | Beyond Accuracy: Investigating Error Types in GPT-4 Responses to USMLE Questions |
| 27 | 2024 | Kruidering M, Truongb B. B, Willey J. M, et al. | Can Generative Artificial Intelligence (AI) Reliably Score Open-Ended Questions (OEQs) in the Assessment of Medical Knowledge |
| 28 | 2024 | Nakao, T., Miki, S., Abe, O., et al. | Capability of GPT-4V(ision) in the Japanese National Medical Licensing Examination: Evaluation Study |
| 29 | 2024 | Danehy, T., Hecht, J., Jariwala, S. P., et al. | ChatGPT Performs Worse on USMLE-Style Ethics Questions Compared to Medical Knowledge Questions |
| 30 | 2024 | Bicknell, B. T., Butler, D., Dzubinski, L., et al. | ChatGPT-4 Omni performance in USMLE disciplines and clinical skills: Comparative analysis |
| 31 | 2024 | Bajčetić M, Mirčić A, Zaletel, I., et al. | Comparing the performance of artificial intelligence learning models to medical students in solving histology and embryology multiple choice questions |
| 32 | 2024 | Li, D. J., Kao, Y. C., Su, K. P., et al. | Comparing the performance of ChatGPT GPT-4, Bard, and Llama-2 in the Taiwan Psychiatric Licensing Examination and in differential diagnosis with multi-center psychiatrists |
| 33 | 2024 | Zare, S., Vafaeian, S., Golestani, A., et al. | Comparing the performance of ChatGPT-3.5-Turbo, ChatGPT-4, and Google Bard with Iranian students in pre-internship comprehensive exams |
| 34 | 2024 | Abbas, A., Rehman, M. S., Rehman, S. S., et al. | Comparing the Performance of Popular Large Language Models on the National Board of Medical Examiners Sample Questions |
| 35 | 2024 | Jaworski, A., Jasiński D, Jasińska N, et al. | Comparison of the Performance of Artificial Intelligence Versus Medical Professionals in the Polish Final Medical Examination |
| 36 | 2024 | Meyer, A., Riese, J., Streichert, T., et al. | Comparison of the Performance of GPT-3.5 and GPT-4 With That of Medical Students on the Written German Medical Licensing Examination: Observational Study |
| 37 | 2024 | Roos, J., Martin, R., Kaczmarczyk, R., et al. | Evaluating Bard Gemini Pro and GPT-4 Vision Against Student Performance in Medical Visual Question Answering: Comparative Case Study |
| 38 | 2024 | Farhat, F., Chaudhry, B. M., Madsen DØ, et al. | Evaluating Large Language Models for the National Premedical Exam in India: Comparative Analysis of GPT-3.5, GPT-4, and Bard |
| 39 | 2024 | Patel, D., Raut, G., Timsina, P., et al. | Evaluating prompt engineering on GPT-3.5’s performance in USMLE-style medical calculations and clinical scenarios generated by GPT-4 |
| 40 | 2024 | Kato, Y., Ushida, K., Momosaki, R., et al. | Evaluating the Accuracy of ChatGPT in the Japanese Board-Certified Physiatrist Examination |
| 41 | 2024 | Hadi, A., Tran, E., Kirpalani, A., et al. | Evaluation of ChatGPT as a diagnostic tool for medical learners and clinicians |
| 42 | 2024 | Rojas, M., Rojas, M., Salehi, S., et al. | Exploring the Performance of ChatGPT Versions 3.5, 4, and 4 With Vision in the Chilean Medical Licensing Examination: Observational Study |
| 43 | 2024 | Booth, G. J., Hauert, T., Moore, J., et al. | Fine-Tuning Large Language Models to Enhance Programmatic Assessment in Graduate Medical Education |
| 44 | 2024 | Kipp, M., Kipp, M., et al. | From GPT-3.5 to GPT-4.o: A Leap in AI’s Medical Exam Performance |
| 45 | 2024 | Ebel, S., Ehrengut, C., Beeskow, A. B., et al. | GPT-4o’s competency in answering the simulated written European Board of Interventional Radiology exam compared to a medical student and experts in Germany and its ability to generate exam items on interventional radiology: a descriptive study |
| 46 | 2024 | Ming, S., Guo, Q., Lei, B., et al. | Influence of Model Evolution and System Roles on ChatGPT’s Performance in Chinese Medical Licensing Exams: Comparative Study |
| 47 | 2024 | Cerame, A., Juaneda, J., Carrasco, J. P., et al. | Is GPT-4 capable of passing MIR 2023? Comparison between GPT-4 and ChatGPT-3 in the MIR 2022 and 2023 exams |
| 48 | 2024 | Morreel, S., Verhoeven, V., Mathysen, D., et al. | Microsoft Bing outperforms five other generative artificial intelligence chatbots in the Antwerp University multiple choice medical license exam |
| 49 | 2024 | Choi, Y. K., Lin, S. Y., Santoso, K., et al. | Optimizing ChatGPT's Interpretation and Reporting of Delirium Assessment Outcomes: Exploratory Study |
| 50 | 2024 | Wang, H. L., Zhou, H., Ye, Z. W., et al. | Performance Assessment of GPT 4.0 on the Japanese Medical Licensing Examination |
| 51 | 2024 | Chen, Y., Huang, X., Li, X., et al. | Performance of ChatGPT and Bard on the medical licensing examinations varies across different cultures: a comparison study |
| 52 | 2024 | Zong, H., Li, J., Shen, B., et al. | Performance of ChatGPT on Chinese national medical licensing examinations: a five-year examination evaluation study for physicians, pharmacists and nurses |
| 53 | 2024 | Huang, C. H., Hsiao, H. J., Kao, C. H., et al. | Performance of ChatGPT on Stage 1 of the Taiwanese medical licensing exam |
| 54 | 2024 | Gandhi, A. P., Joesph, F. K., Behera, A., et al. | Performance of ChatGPT on the India Undergraduate Community Medicine Examination: Cross-Sectional Study |
| 55 | 2024 | Miyazaki, Y., Hata, M., Ikeda, M., et al. | Performance of ChatGPT-4o on the Japanese Medical Licensing Examination: Evalution of Accuracy in Text-Only and Image-Based Questions |
| 56 | 2024 | Hersh, W., Fultz Hollis, K., Fultz Hollis, K., et al. | Results and implications for generative AI in a large introductory biomedical and health informatics course |
| 57 | 2024 | Khake A, Khake S, Desai M, et al. | ROLE OF CHAT GPT 3.5 AND BARD AS SELF ASSESSMENT TOOL FOR UNDERGRADUATE LEVEL MULTIPLE CHOICE QUESTIONS IN OPHTHALMOLOGY |
| 58 | 2024 | Sabaner, M. C., Hashas, A. S. K., Subhi, Y., et al. | The performance of artificial intelligence-based large language models on ophthalmology-related questions in Swedish proficiency test for medicine: ChatGPT-4 omni vs Gemini 1.5 Pro |
| 59 | 2024 | Takagi, S., Koda, M., Watari, T., et al. | The Performance of ChatGPT-4V in Interpreting Images and Tables in the Japanese Medical Licensing Exam |
| 60 | 2025 | Bulut, B., Öz, M. A., Kocaaslan, R., et al. | A comparative analysis of the performance of large language models in the basic life support exam: Comprehensive evaluation of ChatGPT-4o, Gemini 2.0, Claude 3.5, and DeepSeek R1 |
| 61 | 2025 | Mishra, V., Lurie, Y., Mark, S., et al. | Accuracy of LLMs in medical education: evidence from a concordance test with medical teacher |
| 62 | 2025 | Fares, M. Y., Parmar, T., Abboud, J. A., et al. | An Assessment of the Performance of Different Chatbots on Shoulder and Elbow Questions |
| 63 | 2025 | Alfertshofer, M., Knoedler, S., Pomahac, B., et al. | Analyzing Question Characteristics Influencing ChatGPT’s Performance in 3000 USMLE®-Style Questions |
| 64 | 2025 | Ng, O., Phua, D. H., Cleland, J., et al. | Answering Patterns in SBA Items: Students, GPT3.5, and Gemini |
| 65 | 2025 | Salman, I. M., Ameer, O. Z., Hsieh, Y.-H., et al. | Artificial intelligence in healthcare education: evaluating the accuracy of ChatGPT, Copilot, and Google Gemini in cardiovascular pharmacology |
| 66 | 2025 | Casals-Farre, O., Baskaran, R., Hassoulas, A., et al. | Assessing ChatGPT 4.0's Capabilities in the United Kingdom Medical Licensing Examination (UKMLA): A Robust Categorical Analysis |
| 67 | 2025 | Urda-Cîmpean AE, Leucuța DC, Drugan, T., et al. | Assessing the Accuracy of Diagnostic Capabilities of Large Language Models |
| 68 | 2025 | Mehta, S., Haddad, E. N., Mehta, N., et al. | Assessment of Large Language Model Performance on Medical School Essay-Style Concept Appraisal Questions: Exploratory Study |
| 69 | 2025 | Heo, W. Y., Park, H. D., Park, H. D., et al. | Assessment of large language models in medical quizzes for clinical chemistry and laboratory management: implications and applications for healthcare artificial intelligence |
| 70 | 2025 | Wraith, C., Carnegy, A., Sam, A. H., et al. | Can educators distinguish between medical student and generative AI-authored reflections? |
| 71 | 2025 | McBride, E., Leung, E., Ford, J., et al. | Can medical students use artificial intelligence to learn transfusion? Evaluating ChatGPT responses to the American Society of Hematology medical student transfusion learning objectives |
| 72 | 2025 | Roy, R. D., Gupta, S. D., Chowdhury, P. D., et al. | Chat GPT, Gemini or Meta AI: A comparison of AI platforms as a tool for answering higher-order questions in microbiology |
| 73 | 2025 | Cheng, H. Y., Cheng, H. Y., et al. | ChatGPT's Attitude, Knowledge, and Clinical Application in Geriatrics Practice and Education: Exploratory Observational Study |
| 74 | 2025 | Keshtkar, A., Hayat, A. A., Hashempur, M. H., et al. | ChatGPT's Performance on Iran's Medical Licensing Exams |
| 75 | 2025 | Prazeres, F., Prazeres, F., et al. | ChatGPT's Performance on Portuguese Medical Examination Questions: Comparative Analysis of ChatGPT-3.5 Turbo and ChatGPT-4o Mini |
| 76 | 2025 | Ignjatović A, Anđelković Apostolović M, Otašević S, et al. | ChatGPT's progress over time: A longitudinal enhancing biostatistical problem-solving in medical education |
| 77 | 2025 | Mavrych, V., Yaqinuddin, A., Bolgova, O., et al. | Claude, ChatGPT, Copilot, and Gemini performance versus students in different topics of neuroscience |
| 78 | 2025 | Recinella G, Altini C, Maestri L, et al. | Comparative accuracy of ChatGPT-o1, DeepSeek R1, and Gemini 2.0 in answering general primary care questions |
| 79 | 2025 | Bolgova, O., Ganguly, P., Mavrych, V., et al. | Comparative analysis of LLMs performance in medical embryology: A cross-platform study of ChatGPT, Claude, Gemini, and Copilot |
| 80 | 2025 | Singal, A., Goyal, S., Goyal, S., et al. | Comparative evaluation of AI platforms “Google Gemini 2.5 Flash, Google Gemini 2.0 Flash, DeepSeek V3 and ChatGPT 4o” in solving multiple-choice questions from different subtopics of anatomy |
| 81 | 2025 | Szabo, A., Laein, G. D., Laein, G. D., et al. | Comparative evaluation of large language models performance in medical education using urinary system histology assessment |
| 82 | 2025 | Shah, P., Boughanem, D. J., Hanna, K., et al. | Comparative Performance of Large Language Models in Muscle Histology Classification Highlights Enhanced Accuracy of ChatGPT-4o in Tissue Identification |
| 83 | 2025 | Rodrigues Alessi, M., Gomes, H. A., Okamoto, C., et al. | Comparative Performance of Medical Students, ChatGPT-3.5 and ChatGPT-4.0 in Answering Questions From a Brazilian National Medical Exam: Cross-Sectional Questionnaire Study |
| 84 | 2025 | Zhang, R., Cai, Q., Collette, H., et al. | Comparing Artificial Intelligence Large Language Models in Medical Training: A Performance Analysis of ChatGPT and DeepSeek on United States Medical Licensing Examination (USMLE) Style Questions |
| 85 | 2025 | Salvador, R., Vas, D., Nicolau, C., et al. | Comparing ChatGPT and medical student performance in a real image-based Radiology and Applied Physics in Medicine exam |
| 86 | 2025 | Gerard, A. O., Merino, D., Fournier, J.-P., et al. | Evaluating and leveraging large language models in clinical pharmacology and therapeutics assessment: From exam takers to exam shapers |
| 87 | 2025 | Yang, Q.-C., Zeng, Y.-M., Shao, Y., et al. | Evaluating multiple large language models on orbital diseases |
| 88 | 2025 | Agarwal, M., Sharma, P., Wani, P., et al. | Evaluating the Accuracy and Reliability of Large Language Models (ChatGPT, Claude, DeepSeek, Gemini, Grok, and Le Chat) in Answering Item-Analyzed Multiple-Choice Questions on Blood Physiology |
| 89 | 2025 | Liu, M., Okuhara, T., Kiuchi, T., et al. | Evaluating the Effectiveness of advanced large language models in medical Knowledge: A Comparative study using Japanese national medical examination |
| 90 | 2025 | Luo, D., Liu, M., Zheng, Z., et al. | Evaluating the performance of GPT-3.5, GPT-4, and GPT-4o in the Chinese National Medical Licensing Examination |
| 91 | 2025 | Meo, S. A., Abukhalaf, F. A., Sattar, K., et al. | Exploring the role of DeepSeek-R1, ChatGPT-4, and Google Gemini in medical education: How valid and reliable are they? |
| 92 | 2025 | Kaewboonlert, N., Poontananggul, J., Bhakdisongkhram, G., et al. | Factors Associated With the Accuracy of Large Language Models in Basic Medical Science Examinations: Cross-Sectional Study |
| 93 | 2025 | Pérez Moya, F., Pérez Moya, F., et al. | Generative Artificial Intelligence: Capabilities of Llama-3.1-70B-Instruct in Biochemistry and Metabolism |
| 94 | 2025 | Kawahara, T., Sumi, Y., Sumi, Y., et al. | GPT-4/4V's performance on the Japanese National Medical Licensing Examination |
| 95 | 2025 | Krumsvik, R. J., Krumsvik, R. J., et al. | GPT-4's capabilities for formative and summative assessments in Norwegian medicine exams-an intrinsic case study in the early phase of intervention |
| 96 | 2025 | Lee, Z.-W., Yang, L., Ang, J. Y. H., et al. | How Well Can an LLM Chatbot Clarify Queries by Medical Students in Biomedical TBL? |
| 97 | 2025 | Hsieh, M. Y., Wang, T. L., Chou, M. C., et al. | Impact of Prompt Engineering on the Performance of ChatGPT Variants Across Different Question Types in Medical Student Examinations |
| 98 | 2025 | Bolgova, O., Shypilova, I., Mavrych, V., et al. | Large Language Models in Biochemistry Education: Comparative Evaluation of Performance |
| 99 | 2025 | Mavrych, V., Yousef, E. M., Bolgova, O., et al. | Large language models in medical education: a comparative cross-platform evaluation in answering histological questions |
| 100 | 2025 | Cadiente, A., Chen, J., Pilkington, B., et al. | Large Language Models Take on the AAMC Situational Judgment Test: Evaluating Dilemma-Based Scenarios |
| 101 | 2025 | Bessa, R. F., de Oliveira, A. C., Teles, A. S., et al. | Performance Comparison of Large Language Models on Brazil’s Medical Revalidation Exam for Foreign-Trained Graduates |
| 102 | 2025 | Banerjee, A., Chatterjee, M., Sarangi, P. K., et al. | Performance of ChatGPT-3.5 and ChatGPT-4 in Solving Questions Based on Core Concepts in Cardiovascular Physiology |
| 103 | 2025 | Wu, J., Wang, Z. H., Qin, Y. F., et al. | Performance of DeepSeek-R1 and ChatGPT-4o on the Chinese National Medical Licensing Examination: A Comparative Study |
| 104 | 2025 | Saowaprut, P., Wabina, R. S., Siriwat, L., et al. | Performance of large language models on Thailand’s national medical licensing examination: a cross-sectional study |
| 105 | 2025 | Madrid, J., Diehl, P., Benning, L., et al. | Performance of Plug-In Augmented ChatGPT and Its Ability to Quantify Uncertainty: Simulation Study on the German Medical Board Examination |
| 106 | 2025 | Yang, X., Chen, W., Chen, W., et al. | The performance of ChatGPT on medical image-based assessments and implications for medical education |
| 107 | 2025 | Sharma, P., Thapa, K., Filipe, V., et al. | Unlocking the Potential of Large Language Models for AI-Assisted Medical Education: A Case Study with ChatGPT |
| 108 | 2025 | Yang, Z. C., Yao, Z. H., Yu, H., et al. | Unveiling GPT-4V's hidden challenges behind high accuracy on USMLE questions: Observational Study |
| 109 | 2025 | Mavrych, V., Ganguly, P., Bolgova, O., et al. | Using large language models (ChatGPT, Copilot, PaLM, Bard, and Gemini) in Gross Anatomy course: Comparative analysis |
| 110 | 2025 | Chen, T. A., Lin, K. C., Chen, T. J., et al. | While GPT-3.5 is unable to pass the Physician Licensing Exam in Taiwan, GPT-4 successfully meets the criteria |

**Level 0.1: No intervention for students (n=119)**

**(awareness of GenAI, GenAI as content generator etc.)**

| **LP** | **year** | **authors** | **title** |
| --- | --- | --- | --- |
| 1 | 2023 | Cross, J., Robinson, R., Sebastian, R., et al. | Transforming Medical Education: Assessing the Integration of ChatGPT Into Faculty Workflows at a Caribbean Medical School |
| 2 | 2023 | Klang, E., Portugez, S., Meiri, H., et al. | Advantages and pitfalls in utilizing artificial intelligence for crafting medical examinations: a medical education pilot study with GPT-4 |
| 3 | 2023 | Masikisiki, B., Marivate, V., Hlophe, Y., et al. | Investigating the Efficacy of Large Language Models in Reflective Assessment Methods through Chain of Thought Prompting |
| 4 | 2023 | Shimizu, I., Kasai, H., Kawakami, E., et al. | Developing Medical Education Curriculum Reform Strategies to Address the Impact of Generative AI: Qualitative Study |
| 5 | 2023 | Tangadulrat, P., Sono, S., Tangtrakulwanich, B., et al. | Using ChatGPT for Clinical Practice and Medical Education: Cross-Sectional Survey of Medical Students’ and Physicians’ Perceptions |
| 6 | 2024 | Amitani M, Suzuki H, Owaki T, et al. | Evaluating the application of AI technology in motivational interviewing and behavior change assessment |
| 7 | 2024 | Bakkum, M. J., Hartjes, M. G., Agtmael, M. A., et al. | Using artificial intelligence to create diverse and inclusive medical case vignettes for education |
| 8 | 2024 | Benboujja, F., Hartnick, E., Hartnick, C., et al. | Overcoming language barriers in pediatric care: a multilingual, AI-driven curriculum for global healthcare education |
| 9 | 2024 | Beyoğlu, M. M., Kaya, E., Karabulut, E., et al. | Assessment of the quality, readability, and usefulness of ChatGPT generated medical information for ten common cancer types |
| 10 | 2024 | Burke, H. B., Hoang, A., Gagarin, V., et al. | Assessing the Ability of a Large Language Model to Score Free-Text Medical Student Clinical Notes: Quantitative Study |
| 11 | 2024 | Cervantes, J., Smith, B., Rajput, V., et al. | Decoding medical educators’ perceptions on generative artificial intelligence in medical education |
| 12 | 2024 | Clauser, B. E., Yaneva, V., Mee, J., et al. | Automated Scoring of Short-Answer Questions: A Progress Report |
| 13 | 2024 | Collins, B. R., Black, E. W., Rarey, K. E., et al. | Introducing AnatomyGPT: A customized artificial intelligence application for anatomical sciences education |
| 14 | 2024 | Contreras, I., Hossfeld, S., Ghidinelli, M., et al. | Revolutionising Faculty Development and Continuing Medical Education Through AI-Generated Videos |
| 15 | 2024 | Deb Roy A, Bharat Jaiswal I, Mondal, H., et al. | Assessing the Utilization of Large Language Model Chatbots for Educational Purposes by Medical Teachers: A Nationwide Survey From India |
| 16 | 2024 | Driesnack, S., Rücker, F., Viehweger, A., et al. | A practice-based approach to teaching antimicrobial therapy using artificial intelligence and gamified learning |
| 17 | 2024 | Ganjavi, C., Eppler, M., Cacciamani, G. E., et al. | ChatGPT and large language models (LLMs) awareness and use. A prospective cross-sectional survey of U.S. medical students |
| 18 | 2024 | Gin, B. C., Ten Cate O, Boscardin, C., et al. | Assessing supervisor versus trainee viewpoints of entrustment through cognitive and affective lenses: an artificial intelligence investigation of bias in feedback |
| 19 | 2024 | Grévisse, C., Grévisse, C., et al. | LLM-based automatic short answer grading in undergraduate medical education |
| 20 | 2024 | Grévisse, C., Pavlou, M. A. S., Schneider, J. G., et al. | Docimological Quality Analysis of LLM-Generated Multiple Choice Questions in Computer Science and Medicine |
| 21 | 2024 | Harinee, S., Vimal Raja, R., Ragavendhiran, T., et al. | Elevating Medical Training: A Synergistic Fusion of AI and VR for Immersive Anatomy Learning and Practical Procedure Mastery |
| 22 | 2024 | Holderried, F., Stegemann-Philipps, C., Mahling, M., et al. | A Language Model-Powered Simulated Patient With Automated Feedback for History Taking: Prospective Study |
| 23 | 2024 | Huang, H., Lin, H. C., Lin, H. C., et al. | ChatGPT as a life coach for professional identity formation in medical education: A self-regulated learning perspective |
| 24 | 2024 | Kaliterna, M., Žuljević, M. F., Duplančić, D., et al. | Testing the capacity of Bard and ChatGPT for writing essays on ethical dilemmas: A cross-sectional study |
| 25 | 2024 | Kıyak, Y. S., Coşkun, Ö, Uluoǧlu, C., et al. | ChatGPT for generating multiple-choice questions: Evidence on the use of artificial intelligence in automatic item generation for a rational pharmacotherapy exam |
| 26 | 2024 | Laupichler, M. C., Rother, J. F., Raupach, T., et al. | Large Language Models in Medical Education: Comparing ChatGPT- to Human-Generated Exam Questions |
| 27 | 2024 | Liu, F., Chang, X., Wang, H., et al. | Assessing clinical medicine students’ acceptance of large language model: based on technology acceptance model |
| 28 | 2024 | Liu, J. Q. J., Hui, K. T. K., Wong, A. Y. L., et al. | The great detectives: humans versus AI detectors in catching large language model-generated medical writing |
| 29 | 2024 | Lotto, C., Sheppard, S. C., Anschuetz, L., et al. | ChatGPT Generated Otorhinolaryngology Multiple-Choice Questions: Quality, Psychometric Properties, and Suitability for Assessments |
| 30 | 2024 | Lucas, P., Michael, F., Julian, V., et al. | Educating the Next Generation: Making Large Language Models Mandatory in Medical Training |
| 31 | 2024 | Maimone, C., Dolan, B. M., O’Brien, C. L., et al. | Using Natural Language Processing to Visualize Narrative Feedback in a Medical Student Performance Dashboard |
| 32 | 2024 | O'Malley, A., Veenhuizen, M., Ahmed, A., et al. | Ensuring Appropriate Representation in Artificial Intelligence-Generated Medical Imagery: Protocol for a Methodological Approach to Address Skin Tone Bias |
| 33 | 2024 | Pan, G., Ni, J., Ni, J., et al. | A cross sectional investigation of ChatGPT-like large language models application among medical students in China |
| 34 | 2024 | Salih, S. M., Salih, S. M., et al. | Perceptions of Faculty and Students About Use of Artificial Intelligence in Medical Education: A Qualitative Study |
| 35 | 2024 | Sallam, M., Al-Mahzoum, K., Barakat, M., et al. | Anxiety among Medical Students Regarding Generative Artificial Intelligence Models: A Pilot Descriptive Study |
| 36 | 2024 | Sauder, M., Tritsch, T., Shoja, M. M., et al. | Exploring Generative Artificial Intelligence-Assisted Medical Education: Assessing Case-Based Learning for Medical Students |
| 37 | 2024 | Sridharan, K., Sequeira, R. P., Sequeira, R. P., et al. | Artificial intelligence and medical education: application in classroom instruction and student assessment using a pharmacology & therapeutics case study |
| 38 | 2024 | Tao, W. J., Yang, J. M., Qu, X., et al. | Utilization of, Perceptions on, and Intention to Use AI Chatbots Among Medical Students in China: National Cross-Sectional Study |
| 39 | 2024 | Xu, A. Y., Piranio, V. S., Brennan, M., et al. | A Pilot Study of Medical Student Opinions on Large Language Models |
| 40 | 2024 | Yanagita, Y., Yokokawa, D., Ikusaka, M., et al. | Expert assessment of ChatGPT’s ability to generate illness scripts: an evaluative study |
| 41 | 2024 | Zhang, J. S., Yoon, C., Pinkas, A., et al. | Exploring the Usage of ChatGPT Among Medical Students in the United States |
|  |  |  |  |
|  |  |  |  |
| 42 | 2025 | Abi-Rafeh, J., Cattelan, L., Nahai, F., et al. | Artificial Intelligence-Generated Educational Curriculum on Facial Neurotoxin Injections: Assessment of Educational Content, Curriculum Structure, and Course Logistics |
| 43 | 2025 | Abouammoh, N., Alhasan, K., Alhaboob, A., et al. | Perceptions and Earliest Experiences of Medical Students and Faculty With ChatGPT in Medical Education: Qualitative Study |
| 44 | 2025 | Abouzeid, E., Wassef, R., Harris, P., et al. | Chatbots’ Role in Generating Single Best Answer Questions for Undergraduate Medical Student Assessment: Comparative Analysis |
| 45 | 2025 | Ahmed, A., Kerr, E., O'Malley A, et al. | Quality assurance and validity of AI-generated single best answer questions |
| 46 | 2025 | Ahmed, W. M. M., Abdalmotalib, M. M., Mohamed, H. O. Y., et al. | Shaping the future of medical education: A cross-sectional study on ChatGPT attitude and usage among medical students in Sudan |
| 47 | 2025 | Alhur, A. A., Khlaif, Z. N., Hussein, E., et al. | Paradox of AI in Higher Education: Qualitative Inquiry Into AI Dependency Among Educators in Palestine |
| 48 | 2025 | Aneesh K. V, Mohanan S, Nair S. S, et al. | Effectiveness of generative ai versus traditional resources for self-directed learning in physiology among mbbs students: a comparative interventional study |
| 49 | 2025 | Arain, S. A., Akhund, S. A., Meo, S. A., et al. | Transforming medical education: leveraging large language models to enhance PBL-a proof-of-concept study |
| 50 | 2025 | Balu, A., Prvulovic, S. T., Keating, G., et al. | Evaluating the value of AI-generated questions for USMLE step 1 preparation: A study using ChatGPT-3.5 |
| 51 | 2025 | Bedi, S., Fleming, S. L., Shah, N. H., et al. | QUEST-AI: A System for Question Generation, Verification, and Refinement using AI for USMLE-Style Exams |
| 52 | 2025 | Birks, S., Gray, J., Darling-Pomranz, C., et al. | Using artificial intelligence to provide a ‘flipped assessment’ approach to medical education learning opportunities |
| 53 | 2025 | Boddepalli D, Solanke B. P, Solanke B. P, et al. | Evaluating the Role of Generative AI and ChatGPT in Pharmacology Education: A Medical Student Perspective |
| 54 | 2025 | Bolgova, O., Ganguly, P., Mavrych, V., et al. | Evaluating large language models as graders of medical short answer questions: a comparative analysis with expert human graders |
| 55 | 2025 | Camarata, T., McCoy, L., Berman, J., et al. | LLM-Generated multiple choice practice quizzes for preclinical medical students |
| 56 | 2025 | Campbell, K. K., Holcomb, M. J., Scott, D. J., et al. | Applying state-of-the-art artificial intelligence to grading in simulation-based education: assessment, feedback, and ROI |
| 57 | 2025 | Cervantes, J., Cervantes, J., et al. | An Introductory Module of Generative Artificial Intelligence in Medical Education |
| 58 | 2025 | Chakri, I., El Khayali O, Lahlou, L., et al. | Knowledge and Perceptions of AI Among Medical Students in Morocco: Cross-Sectional Study |
| 59 | 2025 | Chen, M., Ma, J., Qiao, Y., et al. | Advancing medical education in cervical cancer control with large language models for multiple-choice question generation |
| 60 | 2025 | Ch'en, P. Y., Day, W., Cassese, T., et al. | GPT-4 generated answer rationales to multiple choice assessment questions in undergraduate medical education |
| 61 | 2025 | Choi, D., Jung, Y., Kim, T., et al. | LLM-Based Clinical History Taking System: A Persona-Driven Approach |
| 62 | 2025 | Cole, J., Duncan, J., Cole, R., et al. | Using Pretrained Large Language Models for AI-Driven Assessment in Medical Education |
| 63 | 2025 | Cross, J., Kayalackakom, T., Naik, S., et al. | Assessing ChatGPT’s Capability as a New Age Standardized Patient: Qualitative Study |
| 64 | 2025 | Deng, A., Chen, W., Rao, M., et al. | Current application of ChatGPT in undergraduate nuclear medicine education: Taking Chongqing Medical University as an example |
| 65 | 2025 | Dinc, M. T., Bardak, A. E., Noronha, C., et al. | Comparative analysis of large language models in clinical diagnosis: Performance evaluation across common and complex medical cases |
| 66 | 2025 | Duan, S., Liu, C., Liu, B., et al. | Integrating AI in medical education: a comprehensive study of medical students’ attitudes, concerns, and behavioral intentions |
| 67 | 2025 | Elabd, N., Rahman, Z. M., Baltatu, O. C., et al. | Designing Personalized Multimodal Mnemonics With AI: A Medical Student's Implementation Tutorial |
| 68 | 2025 | ELdesoqui, M., al-Badawi, E. A., Elsaid, M. A. E., et al. | Assessing the Anatomical Accuracy of AI-Generated Medical Illustrations: A Comparative Study of Text-to-Image Generator Tools in Anatomy Education |
| 69 | 2025 | Elhassan, S. E., Sajid, M. R., Tamim, H., et al. | Assessing Familiarity, Usage Patterns, and Attitudes of Medical Students Toward ChatGPT and Other Chat-Based AI Apps in Medical Education: Cross-Sectional Questionnaire Study |
| 70 | 2025 | Elzayyat, M., Mohammad, J. N., Zaqout, S., et al. | Assessing LLM-generated vs. expert-created clinical anatomy MCQs: a student perception-based comparative study in medical education |
| 71 | 2025 | Furey, M. J., Stemrich, R., Hoffman, R. L., et al. | Can Artificial Intelligence Coach Faculty to Utilize Growth Mindset Language? A Qualitative Analysis of Feedback Statements |
| 72 | 2025 | García-Rudolph A, Sanchez-Pinsach, D., Vidal, J., et al. | Assessing readability of explanations and reliability of answers by GPT-3.5 and GPT-4 in non-traumatic spinal cord injury education |
| 73 | 2025 | Geathers, J., Hicke, Y., Shung, D., et al. | Benchmarking Generative AI for Scoring Medical Student Interviews in Objective Structured Clinical Examinations (OSCEs) |
| 74 | 2025 | Geng, W., Jiang, Y., Shi, L., et al. | Can artificial intelligence read between the lines: Utilizing ChatGPT to evaluate medical students’ implicit attitudes towards doctor–patient relationship |
| 75 | 2025 | Ghaffari, F., Langarizadeh, M., Saberi, M., et al. | Effectiveness of ChatGPT for Clinical Scenario Generation: A Qualitative Study |
| 76 | 2025 | Ghanem, O. A., Hagag, A. M., Hamed, M. S., et al. | Medical students’ knowledge, attitudes, and practices toward generative artificial intelligence in Egypt 2024: a Cross-Sectional study |
| 77 | 2025 | Gim, H., Cook, B., Bacchi, S., et al. | Large language model-supported interactive case-based learning: a pilot study |
| 78 | 2025 | Gorcilov J, Lim M, Bacchi S, et al. | Large Language Models Deliver Interactive Learning Cases Accurately and With Rapid Appropriate Feedback |
| 79 | 2025 | Güvel, M. C., Kiyak, Y. S., Uluoglu, C., et al. | Generative AI vs. human expertise: a comparative analysis of case-based rational pharmacotherapy question generation |
| 80 | 2025 | Haider, S. A., Prabha, S., Forte, A. J., et al. | A Validity Analysis of Text-to-Image Generative Artificial Intelligence Models for Craniofacial Anatomy Illustration |
| 81 | 2025 | Hao, P., Wang, H., Zhu, L., et al. | Enhancing Visual Reasoning with LLM-Powered Knowledge Graphs for Visual Question Localized-Answering in Robotic Surgery |
| 82 | 2025 | Harris, E. C., Baldwin, A., Sperr, E. V., et al. | Surveying Health Professions Students’ Use of Generative AI Applications |
| 83 | 2025 | Hu, H., Wallace, D. D., Boateng, B., et al. | Medical Education Learning Specialists in the Age of Artificial Intelligence |
| 84 | 2025 | Huh, C. Y., Lee, J., Kim, S.-J., et al. | Using Large Language Models to Simulate History Taking: Implications for Symptom-Based Medical Education |
| 85 | 2025 | Iliyasu, Z., Abdullahi, H. O., Aliyu, M. H., et al. | Correlates of Medical and Allied Health Students' Engagement with Generative AI in Nigeria |
| 86 | 2025 | Jiang, Z., Feng, S., Feng, S., et al. | UsmleGPT: An AI application for developing MCQs via multi-agent system |
| 87 | 2025 | Kang S, Holcomb M. J, Jamieson A. R, et al. | Automatic Physical Examination Segmentation within Objective Structured Clinical Examination Videos |
| 88 | 2025 | Kawakami, C., Nomura, O., Saiki, T., et al. | Rapidly Rising Preference among New-entry Medical Students for Using Generative Artificial Intelligence in Reflective Reports |
| 89 | 2025 | Lam, G., Shammoon, Y., Sam, A. H., et al. | Utility of large language models for creating clinical assessment items |
| 90 | 2025 | Li, R., Wu, T., Wu, T., et al. | Application of Artificial Intelligence Generated Content in Medical Examinations |
| 91 | 2025 | Liu, C. X., Zheng, J. N., Liu, C. J., et al. | Potential to perpetuate social biases in health care by Chinese large language models: a model evaluation study |
| 92 | 2025 | Lundgren, H., Papanagnou, D., Marsick, V. J., et al. | From research to resources: developing a case-based learning curriculum for navigating clinical uncertainty |
| 93 | 2025 | Luordo, D., Arrese, M. T., Garrido, M. V. V., et al. | Application of Artificial Intelligence as an Aid for the Correction of the Objective Structured Clinical Examination (OSCE) |
| 94 | 2025 | Mccoy, L., Ganesan, N., Swaim, M. C., et al. | A Training Needs Analysis for AI and Generative AI in Medical Education: Perspectives of Faculty and Students |
| 95 | 2025 | McLin, R., Croniger, C. M., Wilson-Delfosse, A. L., et al. | GenAI and Faculty Collaboration Support Feasible Development of Curriculum-Aligned Open-Access Board-Style Questions |
| 96 | 2025 | Mehta, N., Nielsen, C., Isaacson, J. H., et al. | Creating custom GPTs for faculty development: An example using the Johari Window and Crucial Conversation frameworks for providing feedback to struggling students |
| 97 | 2025 | Misgav, K., Neufeld-Kroszynski, G., Karnieli-Miller, O., et al. | Human Analysis vs. Artificial Intelligence: Analyzing of Qualitative Medical Students' Narratives |
| 98 | 2025 | Mondal, H., Karri, J. K. K., Gupta, P., et al. | A qualitative survey on perception of medical students on the use of large language models for educational purposes |
| 99 | 2025 | Mondal, H., Mondal, H., et al. | Evolving resource use for self-directed learning in physiology among first-year medical students in a classroom setting |
| 100 | 2025 | Neittaanmäki-Perttu, N., Neittaanmäki-Perttu, N., et al. | Swedish medical students’ attitudes toward artificial intelligence and effects on career plans: a survey |
| 101 | 2025 | Ng, O., Han, S. P., Lee, Z.-W., et al. | Student Perspective Matters for GenAI in Question Setting in Medical Education |
| 102 | 2025 | OuYang, Y., Gao, W., Zeng, Y., et al. | MEDSQ: Towards personalized medical education via multi-form interaction guidance |
| 103 | 2025 | Ouyang, Y., Xu, Y., Li, Q., et al. | CaseMaster: Designing a Probe for Oral Case Presentation Training with LLM Assistance |
| 104 | 2025 | Qian, C., Gao, C., Bacchi, S., et al. | Use of Large Language Models for Rapid Quantitative Feedback in Case-Based Learning: A Pilot Study |
| 105 | 2025 | Rajan, A., Alexander, S. M., Shenvi, C. L., et al. | Can AI grade like a professor? comparing artificial intelligence and faculty scoring of medical student short-answer clinical reasoning exams |
| 106 | 2025 | Roselló Atanet, I., Tomova, M., März, M., et al. | Generating learning guides for medical education with LLMs and statistical analysis of test results |
| 107 | 2025 | Seneviratne, H. M. T. W., Manathunga, S. S., Manathunga, S. S., et al. | Artificial intelligence assisted automated short answer question scoring tool shows high correlation with human examiner markings |
| 108 | 2025 | Shindo, N., Uto, M., Uto, M., et al. | Virtual Simulated Patients for Medical Interviews Using Large Language Models with a Self-refinement Mechanism to Suppress Excessive Responses |
| 109 | 2025 | Sreedhar, R., Chang, L., Park, Y. S., et al. | Comparing Scoring Consistency of Large Language Models with Faculty for Formative Assessments in Medical Education |
| 110 | 2025 | Waldock, W. J., Lam, G., Sam, A. H., et al. | Which curriculum components do medical students find most helpful for evaluating AI outputs? |
| 111 | 2025 | Wang, C., Li, S., Yin, R., et al. | Application of Large Language Models in Medical Training Evaluation-Using ChatGPT as a Standardized Patient: Multimetric Assessment |
| 112 | 2025 | Wang, R., Ding, Y., Gao, Z., et al. | Comparative Evaluation of Teaching Plans on Prostate Cancer Generated by Various Large Language Models and a Human Expert |
| 113 | 2025 | Wei, H., Qiu, J., Yuan, W., et al. | MEDCO: Medical Education Copilots Based on a Multi-agent Framework |
| 114 | 2025 | Weisman, D., Sugarman, A., Comulada, W. S., et al. | Development of a GPT-4-Powered Virtual Simulated Patient and Communication Training Platform for Medical Students to Practice Discussing Abnormal Mammogram Results With Patients: Multiphase Study |
| 115 | 2025 | Wu, H., Lee, D., Palmer, E., et al. | A comparison of the psychometric properties of GPT-4 versus human novice and expert authors of clinically complex MCQs in a mock examination of Australian medical students |
| 116 | 2025 | Wu, H., Su, Z., Ye, J., et al. | Enhancing diabetic retinopathy query responses: assessing large language model in ophthalmology |
| 117 | 2025 | Yapıcı Coşkun, Z., Kıyak, Y. S., Özdemir, Ö, et al. | Large language models for generating script concordance test in obstetrics and gynecology: ChatGPT and Claude |
| 118 | 2025 | Yi, Y., Kim, K.-J., Kim, K.-J., et al. | The feasibility of using generative artificial intelligence for history taking in virtual patients |
| 119 | 2026 | Mohamed, A., Al-Barazie, R., Al-Barazie, R., et al. | Assessing the Potential of AI-Generated Assessments in Medical Education: A Study on Diagnostic Microbiology Using Copilot and Gemini |
