# Supplementary material for: The Forgotten Student—Generative AI Use in Medical Education and the Technology-Integrated Educational Design (TIED) Road Map: Scoping Review
Source: J Med Internet Res. 2026 Oct 15;28:e96099. doi: 10.2196/96099 (PMC13671686; doi:10.2196/96099)
Supplement: Supplementary file 4 [file jmir-v28-e96099-s004.docx]

| **SECTION** | **ITEM** | **PRISMA-ScR CHECKLIST ITEM** | **REPORTED ON PAGE #** |
| --- | --- | --- | --- |
| **TITLE** | | | |
| Title | 1 | Identify the report as a scoping review. | Title page, page 1 |
| **ABSTRACT** | | | |
| Structured summary | 2 | Provide a structured summary that includes (as applicable): background, objectives, eligibility criteria, sources of evidence, charting methods, results, and conclusions that relate to the review questions and objectives. | Abstract; pages 2 and 3 |
| **INTRODUCTION** | | | |
| Rationale | 3 | Describe the rationale for the review in the context of what is already known. Explain why the review questions/objectives lend themselves to a scoping review approach. | Introduction, pages 4-5 |
| Objectives | 4 | Provide an explicit statement of the questions and objectives being addressed with reference to their key elements (e.g., population or participants, concepts, and context) or other relevant key elements used to conceptualize the review questions and/or objectives. | Introduction, page 5, lines 87-97 |
| **METHODS** | | | |
| Protocol and registration | 5 | Indicate whether a review protocol exists; state if and where it can be accessed (e.g., a Web address); and if available, provide registration information, including the registration number. | The protocol for this study has not been published nor formally registered. |
| Eligibility criteria | 6 | Specify characteristics of the sources of evidence used as eligibility criteria (e.g., years considered, language, and publication status), and provide a rationale. | No filtering on year, language or publication status was used: Methods, pages 10, lines 200-202 |
| Information sources* | 7 | Describe all information sources in the search (e.g., databases with dates of coverage and contact with authors to identify additional sources), as well as the date the most recent search was executed. | Databases listed in Search strategy, study eligibility and selection: page 10, Appendix 2 – search strategy |
| Search | 8 | Present the full electronic search strategy for at least 1 database, including any limits used, such that it could be repeated. | Search string included in Search strategy, study eligibility and selection: page 10; Appendix 2 – search strategy |
| Selection of sources of evidence† | 9 | State the process for selecting sources of evidence (i.e., screening and eligibility) included in the scoping review. | Screening process and eligibility criteria described in Search strategy, study eligibility and selection: pages 10-11 |
| Data charting process‡ | 10 | Describe the methods of charting data from the included sources of evidence (e.g., calibrated forms or forms that have been tested by the team before their use, and whether data charting was done independently or in duplicate) and any processes for obtaining and confirming data from investigators. | Methods: Data extraction, analysis and synthesis, pages 11-12 |
| Data items | 11 | List and define all variables for which data were sought and any assumptions and simplifications made. | Methods: Data extraction, analysis and synthesis, pages 11-12 |
| Critical appraisal of individual sources of evidence§ | 12 | If done, provide a rationale for conducting a critical appraisal of included sources of evidence; describe the methods used and how this information was used in any data synthesis (if appropriate). | No quality appraisal has been conducted. |
| Synthesis of results | 13 | Describe the methods of handling and summarizing the data that were charted. | Methods: Data extraction, analysis and synthesis, pages 11-12. |
| **RESULTS** | | | |
| Selection of sources of evidence | 14 | Give numbers of sources of evidence screened, assessed for eligibility, and included in the review, with reasons for exclusions at each stage, ideally using a flow diagram. | Figure 1 |
| Characteristics of sources of evidence | 15 | For each source of evidence, present characteristics for which data were charted and provide the citations. | Study characteristics, page 14; Table 1 and Appendix 3 and 4 |
| Critical appraisal within sources of evidence | 16 | If done, present data on critical appraisal of included sources of evidence (see item 12). | N/A |
| Results of individual sources of evidence | 17 | For each included source of evidence, present the relevant data that were charted that relate to the review questions and objectives. | Table 1 and Appendix 3 and 4 |
| Synthesis of results | 18 | Summarize and/or present the charting results as they relate to the review questions and objectives. | Results, pages: 14-24 |
| **DISCUSSION** | | | |
| Summary of evidence | 19 | Summarize the main results (including an overview of concepts, themes, and types of evidence available), link to the review questions and objectives, and consider the relevance to key groups. | Primary findings, page 25 |
| Limitations | 20 | Discuss the limitations of the scoping review process. | Limitations, pages: 30 |
| Conclusions | 21 | Provide a general interpretation of the results with respect to the review questions and objectives, as well as potential implications and/or next steps. | Comparison with prior work, pages 25-30  Conclusions, page 31 |
| **FUNDING** | | | |
| Funding | 22 | Describe sources of funding for the included sources of evidence, as well as sources of funding for the scoping review. Describe the role of the funders of the scoping review. | Founding section: page 32 |

JBI = Joanna Briggs Institute; PRISMA-ScR = Preferred Reporting Items for Systematic reviews and Meta-Analyses

**PRISMA-S Checklist**

| **Section/topic** | **#** | **Checklist item** | **Location(s) Reported** |
| --- | --- | --- | --- |
| **INFORMATION SOURCES AND METHODS** | | | |
| Database name | 1 | Name each individual database searched, stating the platform for each. | page 10, Appendix 2 – Search strategy: Ovid (Medline, Embase), PubMed, Web of Science, Scopus |
| Multi-database searching | 2 | If databases were searched simultaneously on a single platform, state the name of the platform, listing all of the databases searched. | N/A |
| Study registries | 3 | List any study registries searched. | No additional registries were searched |
| Online resources and browsing | 4 | Describe any online or print source purposefully searched or browsed (e.g., tables of contents, print conference proceedings, web sites), and how this was done. | No additional searches were conducted. |
| Citation searching | 5 | Indicate whether cited references or citing references were examined, and describe any methods used for locating cited/citing references (e.g., browsing reference lists, using a citation index, setting up email alerts for references citing included studies). | Cited references or citing references were not examined. |
| Contacts | 6 | Indicate whether additional studies or data were sought by contacting authors, experts, manufacturers, or others. | Authors, experts or others have not been contacted to sought additional studies |
| Other methods | 7 | Describe any additional information sources or search methods used. | N/A |
| **SEARCH STRATEGIES** | | | |
| Full search strategies | 8 | Include the search strategies for each database and information source, copied and pasted exactly as run. | Methods, Page 10;, Appendix 2 – Search strategy  ("GenAI"[Title/Abstract] OR "generative AI"[Title/Abstract] OR "large language model"[Title/Abstract] OR "gpt"[Title/Abstract] OR "deepseek"[Title/Abstract] OR "LLM"[Title/Abstract]) AND ("medical student"[Title/Abstract] OR "medical education"[Title/Abstract]) |
| Limits and restrictions | 9 | Specify that no limits were used, or describe any limits or restrictions applied to a search (e.g., date or time period, language, study design) and provide justification for their use. | No language, date or publication-type restrictions. |
| Search filters | 10 | Indicate whether published search filters were used (as originally designed or modified), and if so, cite the filter(s) used. | No filters were used |
| Prior work | 11 | Indicate when search strategies from other literature reviews were adapted or reused for a substantive part or all of the search, citing the previous review(s). | Kıyak YS, İş-Kara T, Emekli E. Applications and Outcomes of Large‑Language‑Model‑Generated Feedback in Undergraduate Medical Education: A Scoping Review. MedSciEduc 2026 Jan 6; doi: 10.1007/s40670-025-02621-3  Feigerlova E, Hani H, Hothersall-Davies E. A systematic review of the impact of artificial intelligence on educational outcomes in health professions education. BMC Med Educ 2025 Jan 27;25(1):129. doi: 10.1186/s12909-025-06719-5 |
| Updates | 12 | Report the methods used to update the search(es) (e.g., rerunning searches, email alerts). | None |
| Dates of searches | 13 | For each search strategy, provide the date when the last search occurred. | 22-24 September 2025 |
| **PEER REVIEW** | | | |
| Peer review | 14 | Describe any search peer review process. | The pilot searches were conducted and analyzed collectively by the team to determine agreed approach to precision and recall of the search string. |
| **MANAGING RECORDS** | | | |
| Total Records | 15 | Document the total number of records identified from each database and other information sources. | 2316 |
| Deduplication | 16 | Describe the processes and any software used to deduplicate records from multiple database searches and other information sources. | Deduplication has been done using TERA tools. The tool has also been used to manage the screening process (inclusion, exclusion – Screenatron, and managing conflicts – disputatron). Full text analysis has been documented in MS Excel. |
|  |  |  |  |
|  | | |  |
|  | | |  |
|  | |  |  |
